# Photodynamic Eradication of *Pseudomonas aeruginosa* with Ru-Photosensitizers Encapsulated in Enzyme Degradable Nanocarriers

**DOI:** 10.3390/pharmaceutics15122683

**Published:** 2023-11-27

**Authors:** Kawaljit Kaur, Max Müller, Mareike Müller, Holger Schönherr

**Affiliations:** Physical Chemistry I & Research Center of Micro- and Nanochemistry and (Bio)Technology (*Cμ*), Department of Chemistry and Biology, School of Science and Technology, University of Siegen, 57076 Siegen, Germanymax.mueller@uni-siegen.de (M.M.)

**Keywords:** antimicrobial photodynamic therapy (aPDT), triggered release, *Pseudomonas aeruginosa*, amphiphilic block copolymers, micelles, polymersomes, singlet oxygen, photosensitizers

## Abstract

The development of new approaches for the treatment of the increasingly antibiotic-resistant pathogen *Pseudomonas aeruginosa* was targeted by enhancing the effect of local antimicrobial photodynamic therapy (aPDT) using poly(ethylene glycol)-*block*-poly(lactic acid) (PEG_114_-*block*-PLA_x_) nanocarriers that were loaded with a ruthenium-based photosensitizer (PS). The action of tris(1,10-phenanthroline) ruthenium (II) bis(hexafluorophosphate) (RuPhen3) encapsulated in PEG_114_-*block*-PLA_x_ micelles and vesicles was shown to result in an appreciable aPDT inactivation efficiency against planktonic *Pseudomonas aeruginosa*. In particular, the encapsulation of the PS, its release, and the efficiency of singlet oxygen (^1^O_2_) generation upon irradiation with blue light were studied spectroscopically. The antimicrobial effect was analyzed with two strains of *Pseudomonas aeruginosa*. Compared with PS-loaded micelles, formulations of the PS-loaded vesicles showed 10 times enhanced activity with a strong photodynamic inactivation effect of at least a 4.7 log reduction against both a *Pseudomonas aeruginosa* lab strain and a clinical isolate collected from the lung of a cystic fibrosis (CF) patient. This work lays the foundation for the targeted eradication of *Pseudomonas aeruginosa* using aPDT in various medical application areas.

## 1. Introduction

Antimicrobial resistance (AMR) is among the biggest challenges in modern healthcare. The increasing ineffectiveness of broad spectrum antibiotics, but more importantly also of last-resort antibiotics towards several bacterial pathogens, causes a large number of deaths every year. In the EU, more than 35,000 patients die from AMR-related infections every year, a number comparable to the combined death rate caused by influenza, tuberculosis, and acquired immunodeficiency syndrome (AIDS) [1]. Worldwide, the number of deaths in the context of AMR is estimated to be more than 700,000 per year and is predicted to rise according to the World Health Organization (WHO) to more than 10 million by 2050 [2].

One of the main causes for such an increase in mortality is the limited options for the efficient treatment of antimicrobial-resistant pathogens and also a lack of rapid diagnosis. This results in common nosocomial pathogens, including *Enterococcus faecium*, *Staphylococcus aureus*, *Klebsiella pneumoniae*, *Acinetobacter baumannii*, *Pseudomonas aeruginosa*, and *Enterobacter* spp. (known as the ESKAPE group), becoming drastically increasingly multidrug resistant within the last years [3]. Among the ESKAPE pathogens, *Pseudomonas aeruginosa*, which is also listed as a critical priority 1 pathogen in the WHO’s priority list for the research and development of new antibiotics [4], is one of most common pathogens causing life-threatening respiratory tract infections, for example, in human immunodeficiency virus (HIV) -infected [5] and CF [6] patients. Other common infections caused by this pathogen are diabetic foot, urinary tract, bloodstream, and skin and soft tissue infections [7,8,9]. A big challenge in medicine is the targeted treatment of multidrug-resistant (MDR) and extensively drug-resistant (XDR) *Pseudomonas aeruginosa* that requires susceptibility screening for the available drug classes to circumvent resistance mechanisms [10]. Additionally, tolerance mechanisms due to strong biofilm formation capacity can exacerbate recalcitrant bacterial infections [11].

To treat MDR bacteria, aPDT has been investigated. Due to its fast and efficient bactericidal effect and its lower risk of resistance build-up to a variety of different bacterial pathogens [12], aPDT has been shown to be a promising alternative for the treatment of microbial infections. The principle of aPDT is based on the excitation of PS molecules with light to locally generate reactive oxygen species (ROS), including ^1^O_2_, at the target site. Since the early 1990s, many different systems have been investigated for aPDT, ranging from novel photosensitizers over photoactive surfaces and different kinds of nanoparticles, fibers, and nanocarriers of various origins [12,13,14].

One of the main advantages of aPDT is the localized on-demand treatment of infections caused by MDR bacteria. It has been shown that aPDT possesses a very low risk of contributing to resistance build-up, since the killing of bacteria is based on the action of ROS, which addresses multiple targets in the bacterial cell and hence impedes the resistance build-up [12]. For many common MDR bacteria, aPDT has already been proven to be a strategy to avoid resistance build-up [15,16].

However, many combinatory approaches for aPDT usually only reach a log reduction factor (LRF) < 3. For instance, methylene blue (MB) -loaded electrospun nanofibers showed an LRF of 3 [17], while MB or erythrosine B encapsulated in PCL fibers showed an LRF of only 1 [18]. Inhibitory (not bactericidal) effects were reported for MB in chitosan-loaded silver nanoparticles [19]; PLA nanofibers loaded with indocynine green and curcumin showed a combined LRF > 4 [20]. Various studies have been carried out to improve the efficiency of aPDT using higher irradiation doses or higher PS concentrations to achieve higher LRF values [21,22,23].

Furthermore, different metal-based PSs have also been investigated for aPDT effects for various applications. In 2022, de Siqueira et al. reviewed phthalocyanines containing aluminum, cobalt, gallium, silicon, and zinc as PSs in different nanocarriers, such as liposomes, for studying aPDT against bacteria, fungi, viruses, and protozoans [24]. However, many known PSs used for aPDT show only limited biocompatibility, limited solubility in aqueous or biological media, dark toxicity, and reduced photoactivity in the aggregated state, which leads to difficulties in applications [25,26].

The use of Ru complexes as PSs offers several advantages compared with other PSs. Ru(II) -based complexes have been extensively studied for PDT effects against fatal cancers and some lead complexes undergo phase II clinical studies for PDT treatment of bladder cancer [27,28]. Despite this, challenges with the solubility and stability in aqueous media remain to be improved to achieve optimal performance in medical applications.

One method to overcome such limitations is adapted from the drug delivery of water-insoluble drugs. Among the carriers established for encapsulation approaches are polymeric micelles, polymersomes, liposomes, and different kinds of nanoparticles [29]. Important for such systems is the ability to remain in circulation without passively releasing the payload until reaching the infection site and then releasing the cargo on demand. One method to improve the pharmacokinetics and stabilized circulation within the body is the PEGylation of the nanocarriers, as used in cancer therapy [30,31].

Thus, in this current study, a PEG-based polymer was chosen for the encapsulation of a ruthenium-based photosensitizer in polymeric nanocarriers to increase the solubility and biocompatibility of the PS. Further, the enzyme–labile polymer PLA as the hydrophobic block affords the possibility to potentially release the photosensitizer on demand. In the presence of proteinases, produced by *Pseudomonas aeruginosa* in larger concentrations at the infection site [32], PLA is enzymatically cleaved. Corresponding PLA block copolymers have been previously reported for the encapsulation of signaling molecules and their triggered release in the presence of, e.g., the model enzyme proteinase K [33,34]. In this current study, this strategy was adapted and applied to the encapsulation of a potent Ru-based photosensitizer that was reported to show an aPDT effect against planktonic cultures of *Pseudomonas aeruginosa* and *Staphylococcus aureus* strains [12,35,36]. In particular, the effectiveness of aPDT against *Pseudomonas aeruginosa* was studied for PEG_114_-*block*-PLA_x_ micelles and vesicles (polymersomes).

## 2. Materials and Methods

### 2.1. Materials

Poly(ethylene glycol)methylether (mPEG) (with an average molar mass (*M*_n_) of 5000 g/mol)), tin(II) ethyl hexanoate (92.5–100%), (3S)-cis-3,6-dimethyl-1,4-dioxane-2,5-dione (cis-lactide) (99%), dimethyl sulfoxide (DMSO) 99%, phosphate buffered saline (tablet, 0.01 M, pH 7.4) and Proteinase K from *Tritirachium album*, lyophilized powder, BioUltra ≥ 30 units/mg were purchased from Sigma Aldrich (Taufkirchen, Germany). Tris buffer 100 mM, pH 7.4 was purchased from Merck Millipore (Darmstadt, Germany). Tris(1,10-phenanthroline) ruthenium (II) bis(hexafluorophosphate) (RuPhen3, >98%) was purchased from TCI chemicals (Eschborn, Germany). Deuterated chloroform (99.8 atom-%) was purchased from Deutero (Kastellaun, Germany); tetrahydrofurane (THF, 100%) and methanol (>99.8%)were purchased from VWR (Darmstadt, Germany). Dichloromethane (DCM, 99.8%) was purchased from Fisher Scientific (Schwerte, Germany). Singlet oxygen sensor green (SOSG) and diethyl ether (99.5%) were purchased from Thermo Fischer Scientific (Dreieich, Germany ). Lysogeny broth (LB) (Luria/Miller: 10 g/L tryptone, 5 g/L yeast extract, 10 g/L NaCl, pH 7.0 ± 0.2), LB agar (10 g/L tryptone, 5 g/L yeast extract, 10 g/L NaCl, 15 g/L agar-agar, pH 7.0 ± 0.2), Nile Red (microscopy grade) and the dialysis membrane Spectra/Por^®^ 7 (molecular weight cutoff 50,000 Da) were purchased from Carl Roth GmbH (Karlsruhe, Germany). Sterile water and 0.9% saline was purchased from Versylene Fresenius (Bad Homburg, Germany). Transparent flat-bottom 96-well plate were purchased from Sarstedt (Nümbrecht, Germany) and cell culture 96-black half-area µ-clear-well plate from Greiner Bio-One (Frickenhausen, Germany). Milli-Q water (for preparing LB medium and LB agar plates) was drawn from a Millipore Direct Q8 system with a resistivity of 18.2 MΩ cm, Millipore advantage A10 system with Millimark Express 40 filter (Merck, Darmstadt, Germany).

### 2.2. Methods

#### 2.2.1. Synthesis of PEG_114_-b-PLA_143_ and PEG_114_-b-PLA_499_

The synthesis was adapted from Tücking et al. [34]. Prior to the synthesis, all solid educts were dried overnight in a vacuum oven (5 mbar, at room temperature), and all glassware overnighted in a drying oven at 80 °C. The Schlenk flasks, equipped with stirring bars, were connected to the Schlenk line, repetitively heated under vacuum using a heat gun, filled with argon, and again evacuated to remove remaining moisture and oxygen. All given amounts were exemplary for the synthesis of PEG_114_-*b*-PLA_200_.

mPEG (1.00 g) was added to 0.10 mL stannous octanoate (Sn(oct)_2_) under stirring in an inert argon gas atmosphere at 80 °C. After heating to 130 °C, (3S)-cis-3,6-dimethyl-1,4-dioxane-2,5-dione (cis-lactide) (2.88 g) was added and the mixture was stirred for 30 min at 130 °C. The mixture was then allowed to cool down to room temperature. The obtained solid was dissolved in small amounts of dichloromethane and recrystallized in ice-cold diethyl ether (minimum 10 × volume of DCM). The precipitate was left to settle or was centrifuged at 6000 rpm for 10 min, then the solvent was decanted. The solid was afterwards dissolved and recrystallized a further 4–6 times. The obtained colorless solid was dried overnight in a vacuum oven, ground, dried again, and further characterized by ^1^H-NMR spectroscopy. The recrystallization was continued until the amount of observable monomer was below 1% and the integral ratio of the PLA_x_ protons remained constant relative to the PEG_114_ protons after recrystallization.

#### 2.2.2. Encapsulation of RuPhen3 Photosensitizer into Micelles and Polymersomes Formed from PEG_114_-b-PLA_143_ and PEG_114_-b-PLA_499_ Polymers

An amount of 1.00 mL of the aqueous phase was added, with an addition rate of 0.50 mL/min to a 1.00 mL stirring solution of the respective polymer (3.0–4.0 wt% in THF) and, in case of loaded samples, 5.0 mg of RuPhen3 (Batch1), 10.0 mg of RuPhen3 (Batch2), or 2.0 mg of Nile Red. The turbid or colored mixture was stirred for an additional 10–12 h before 10.00 mL of Milli-Q water was added. The turbid mixture was dialyzed against Milli-Q water, if not described differently (MWCO = 50 kDa), to remove residual polymer and organic solvent. The dialysis was performed for 2 days, and the water was exchanged 3–4 times per day. After dialysis, the dispersion was centrifuged to separate the micelles and polymersomes from the liquid and the supernatant was exchanged with water to remove non-encapsulated PSs. This procedure was repeated until the fluorescence emission of RuPhen3 in the supernatant was below the limit of detection (LOD) in fluorescence spectroscopy.

For batch (B3) with increased volumes, the amounts of used polymer, RuPhen3, and added volumes of water and THF were increased by a factor of 4, respectively, while all other parameters such as concentrations, addition rates, reaction times, and stirring velocity were kept constant. 

#### 2.2.3. Enzymatically Triggered Release of RuPhen3 and Nile Red from PEG_114_-b-PLA_143_ and PEG_114_-b-PLA_499_ Polymers and Fluorescence Spectroscopy

For release experiments, the Nile Red and RuPhen3-loaded nanocontainers were let to settle for at least 1 h to exclude sedimenting agglomerates. An amount of 1.00 mL of dispersion was transferred into a 1.0 cm path-length quartz or polystyrene cuvette, and 1.00 mL of PBS-buffered (0.01 M, pH 7.4) proteinase K solution (5.0 mg/mL) was added. The mixture was homogenized using a pipette and the spectrometer started immediately to monitor the changes in fluorescence emission. The spectra were recorded for a minimum of 12 h, taking one spectrum every 3 min at an excitation wavelength of 530 nm for Nile Red and 450 nm for RuPhen3.

Fluorescence emission spectra were recorded using a Varian Cary Eclipse spectrometer (Mulgrave, Victoria, Australia) at 25 °C using a 1.0 cm path-length quartz cell or 1.0 cm path-length polystyrene cuvettes. Fluorescence spectra were measured at a detector voltage of 1000 V, a scan rate of 120 nm/min, and a resolution of 2.5 nm for the excitation and emission.

For estimation of encapsulated RuPhen3 concentrations in micelle or polymersome dispersions (after removal of non-encapsulated RuPhen3), the fluorescence intensity of RuPhen3 centered around 600 nm (*λ*_ex_ = 450 nm) and respective dilutions were measured and compared to the maximum fluorescence intensity of RuPhen3 solutions with known concentrations. 

#### 2.2.4. Transmission Electron Microscopy and Field Emission Scanning Electron Microscopy 

TEM images were recorded by an FEI Talos F200X transmission electron microscope using an acceleration voltage between 80 and 200 kV. The samples were drop-casted onto copper grids (mesh 400) with ultra-thin carbon film (<3 nm) supported by lacey carbon film (Plano GmbH, Wetzlar, Germany).

For FE-SEM, micelles and polymersome dispersions were drop-casted onto cleaned silicon substrates, which were then dried at room temperature for 24 h, followed by sputtering with a thin gold layer (5–10 nm) in a sputter coater (S150B BOC Edwards, West Sussex, UK) at a pressure of 0.20 mbar in an argon atmosphere for 30 s at a voltage of 1.0 kV. The samples were afterwards placed in a sample holder inside the sample chamber of the FE-SEM. The images were recorded using a field emission scanning electron microscope Zeiss Ultra 55cv (Carl Zeiss, Oberkochen, Germany) at a 5 kV acceleration voltage with the in-lens secondary electron detector. The SmartSEM software (V05.02.05) was used for the collection and processing of the FE-SEM images.

#### 2.2.5. Determination of Mass Concentrations of Nanocarriers in Dispersion 

Mass concentrations of nanocarriers in dispersion were additionally determined by evaporation of dispersant under reduced pressure (100 mbar) inside a vacuum oven, and the weight loss of the used glass vials at an analytical balance after drying for 24 h at 50 °C was measured. The weight of empty vials and the weight of 1.00 mL of dispersions was determined beforehand. This way, the used volume of dispersion and the mass of dried nanocarriers could be determined and the mass concentration calculated.

To confirm the obtained mass, thermogravimetric analysis of single suspension was performed, as described in Section 2.2.8.

#### 2.2.6. ^1^H-NMR Spectroscopy

^1^H-NMR spectra were recorded on a Bruker Avance 400 spectrometer or Jeol ECZ 500 MHz spectrometer. The observed chemical shifts (*δ*) were stated in ppm relative to the solvent signals (CDCl_3_, 1H: *δ* = 7.26 ppm or DMSO-*d*_6_, 1H: δ = 2.50 ppm). The spectra were recorded in glass tubes (Boro400–5–7).

The integral PEG_114_ at 3.64 ppm was normalized to 456 protons, referring to the value of *M*_n_ provided by the supplier. The degree of polymerization of the PLA block was determined by integration of the characteristic peaks around 5.16 and 1.57 ppm, divided by the respective number of protons of the repetition unit. The chemical shifts of the polymer and monomer are stated for the PEG_114_-*b*-PLA_143_ and PEG_114_-*b*-PLA_499_ in deuterated chloroform (CDCl_3_).

PEG_114_-*b*-PLA_143_:

^1^H-NMR (500 MHz, CDCl_3_):*δ* (ppm) = 7.26 (s, CDCl_3_): 5.16 (q, ^3^*J* = J 7.1 Hz, 134H, CH_3_CH(C=O)O (PLA)), 3.64 (s, 456H, OCH_2_CH_2_O (PEG)), 3.36 (s, 3H, OCH_3_) (PEG-OMe), 1.58 (d, ^3^*J* = 7.2 Hz, 437H, CH_3_CH(C=O)O (PLA)), 1.48 (d, ^3^*J* = 7.2 Hz, 6H, CH_3_CH(C=O)O (monomer)) (Figure S1).

PEG_114_-*b*-PLA_499_:

^1^H-NMR (500 MHz, CDCl_3_):*δ* (ppm) = 7.26 (s, CDCl_3_): 5.16 (q, ^3^*J* = J 7.1 Hz, 484H, CH_3_CH(C=O)O (PLA)), 3.64 (s, 456H, OCH_2_CH_2_O (PEG)), 3.36 (s, 3H, OCH_3_) (PEG-OMe), 1.57 (d, ^3^*J* = 7.2 Hz, 1514H, CH_3_CH(C=O)O (PLA)), (Figure S2).

PEG_114_-*b*-PLA_48_:

^1^H-NMR (500 MHz DMSO-*d*_6_) *δ* (ppm) = 5.20 (q, ^3^*J* = 7.0 Hz, 44H, CH_3_CH(C=O)O (PLA)), 5.01 (q, ^3^*J* = J 7.1 Hz,1H, CH_3_CH(C=O)O (monomer)), 3.51 (s, 456H, OCH_2_CH_2_O (PEG)), 3.32 (s, 78H, H_2_O), 3.31 (s, 3H, OCH_3_) (PEG-OMe), 1.46 (d, ^3^*J* = 7.0 Hz, 147 H, CH_3_CH(C=O)O (PLA)) (Figure S3).

#### 2.2.7. Dynamic Light Scattering (DLS)

Size determination by DLS was performed using a Zetasizer Nano (Malvern Instruments Ltd., Worcestershire, UK) at an angle of 173°, backscatter NIBS, using a 4 mW He–Ne laser with a wavelength of 632.8 nm. Each sample was measured at least 3 times; each measurement consisted of 15 scans with a measuring time of 10 s. In case of outliers, the measurement was taken out of the statistics. Between 1.0 and 1.5 mL of dispersion was filled into a disposable polystyrene cuvette and, if needed, filtered using a 400 nm PES syringe filter to remove agglomerates or diluted with Milli-Q water. For PEG_114_-*b*-PLA assemblies, a refractive index of 1.42, with water (*n* = 1.333) as the dispersant, was set for data evaluation with the affiliated Zetasizer Software (V.7.11). 

#### 2.2.8. Thermogravimetric Analysis (TGA)

Thermogravimetric analysis was performed on a TGA Q50 V6.7 Build 203 Universal V4.4A TA Instruments. The sample was dried overnight at 5 mbar, filled into a platinum crucible, placed in the auto sampler of the device, and equilibrated at 50 °C under a nitrogen atmosphere. After equilibration, the temperature was increased to 200 °C (at a rate of 20 °C/min) and from 200 to 450 °C at 5 °C/min. After reaching 450 °C, the temperature was increased further to 600 °C at 20 °C/min. The gas was then changed to oxygen to oxidize remaining organic residues.

#### 2.2.9. Irradiation Experiments

A homemade LED irradiation setup was used to perform the irradiation experiments. The setup included two panels of 225 blue LEDs (14 W, *λ* = 450–460 nm, HQRP LED plant grow panel lamp system, Harrison, NJ, USA) facing each other at adjustable distances. The panels were placed in such a way on the top and bottom to have an exposure area in the center to achieve irradiation from both sides of the well plate. The irradiation intensity at the exposure area was adjusted to 2.7–3.0 mW/cm^2^, as measured using a PM 160 wireless handheld power meter (Thorlabs, Bergkirchen, Germany).

For irradiation, the sample well plate was placed in the exposure area and was irradiated at room temperature twice for 15 min, with 5 min under ambient light exposure between each irradiation. To assess the effect of irradiation, treated samples were compared to samples not exposed to light. In this paper, these conditions are referred to as “OFF” (non-irradiated samples) and “ON” (irradiated samples). 

#### 2.2.10. Bacterial Strains

*Pseudomonas aeruginosa* lab strain ATCC 19660 (isolated from sepsis in Lima, Peru, and purchased from LGC Standards GmbH (Wesel, Germany)) (here noted as PA ATCC) and *Pseudomonas aeruginosa* cystic fibrosis patient strain CH2678 (isolated from sputum, isolated at Charité Berlin, provided by Prof. Susanne Häußler, Helmholtz Centre for Infection Research, Braunschweig, Germany) (denoted here as PA CH2678) were used in this study.

#### 2.2.11. Detection of Singlet Oxygen Production

To detect the production of singlet oxygen, the fluorescent singlet oxygen sensor green SOSG probe was used. In the well plate, 45 µL of the samples was mixed with 45 µL of water or with 3 × 10^6^ ± 2 × 10^6^ CFU/mL bacteria suspension. Afterwards, 10 µL of 100 µM SOSG probe was added (final SOSG concentration: 10 µM). The samples were incubated in the dark for 15 min and then irradiation was performed (see Section 2.2.9). The SOSG fluorescence signal for irradiated and non-irradiated samples was measured at an excitation of 504 nm and emission at 528 nm.

#### 2.2.12. aPDT Effect on Planktonic Bacteria

Overnight cultures were prepared by taking single colonies of each bacterial strain from 5.0 mL LB followed by incubating at 37 °C for 18 h while shaking at 200 rpm. Then, 1.0 mL of the cultures were centrifuged at 10,000 rpm for 5 min. The supernatant was discarded and the pellet was resuspended in 0.9% NaCl. The OD at 600 nm of each bacterial suspension was adjusted to 0.6, corresponding to ~10^8^ CFU/mL. Then the dilution was made in 0.9% NaCl to obtain a final working solution concentration of both bacterial strains of 3 × 10^6^ ± 2 × 10^6^ CFU/mL. Afterward, 25 µL of both bacterial suspensions was distributed in a 96-well plate containing 25 µL of RuPhen3 and RuPhen3-loaded micelles and vesicles. Each sample condition was performed in technical triplicate (3 wells) separately for irradiated and non-irradiated samples. The samples were incubated for 15 min at room temperature and then irradiation was performed. To evaluate the aPDT effect, 5 µL of each technical replicate (well) of the irradiated and non-irradiated undiluted and serially diluted samples (up to 10^5^ fold) was spotted three times on agar plates (15 µL in total), resulting in a detection limit of the method of 67 CFU/mL. The plates were incubated overnight at 37 °C and, the next day, bacteria colonies were counted for CFU/mL calculations of all sample conditions. The log_10_ CFU/mL and the LRF were then calculated. For the LRF, the respective non-irradiated bacteria suspension was taken as the control. The arithmetic mean and the standard deviation were calculated from 3 times independently performed experiments, with each experiment based on 3 technical replicates. 

## 3. Results 

For the study of the encapsulation and release and the aPDT efficiency of the PS RuPhen3, a series of PEG-*b*-PLA block copolymers with different lengths of PLA block was synthesized. As reported in the literature, micelles are typically formed by PEG-*b*-PLA polymers with shorter PLA chains and a mass fraction of PLA below 0.5. The morphology of the assemblies depends nevertheless on the polymer composition and experimental conditions of the assembly process [37,38,39,40]. However, micellar PEG-*b*-PLA structures with PLA fractions above 0.5 and polymersomes with PLA fractions below 0.5 have also been reported [41,42,43]. Thus, polymers with a PLA fraction of more than 0.80 are expected to form polymersomes, while PLA fractions close to 0.5 might lead to formation of either polymersomes or micelles, or even mixtures, depending on experimental conditions. 

### 3.1. Synthesis and Characterization of PEG_114_-b-PLA_143_ and PEG_114_-b-PLA_499_

**Figure 1:**
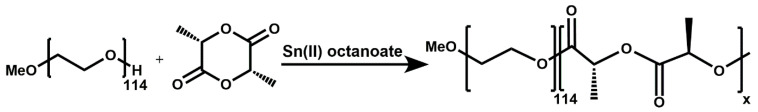
Reaction scheme of the ring-opening polymerization of cis-lactide ((3S)-cis-3,6-Dimethyl-1,4-dioxan-2,5-dion) with PEG_114_-mono methyl ether as macroinitiator in the presence of tin (II) octanoate as a catalyst.

A library of amphiphilic PEG_114_-*b*-PLA polymers with nominal block lengths between 50 and 500 units was synthesized via ring-opening polymerization of cis-lactide according to Figure 1, using the macroinitiator MeO-PEG_114_-OH for PLA, with yields of 82–87%. The synthesized polymers were characterized using ^1^H-NMR spectroscopy and TGA to determine the average number of PLA repeat units and to verify the composition.

The ^1^H-NMR spectra showed the characteristic proton signals for mPEG_114_ and PLA and the residues of the used monomer (cis-lactide), as shown in Figures S1–S7 (Supporting Information). Due to the known unreliability of the gel permeation chromatography (GPC) analysis for this polymer, TGA was used to determine the weight fraction of PEG and PLA of the synthesized polymers. Our GPC results in THF and water differed from the ^1^H-NMR spectroscopy and TGA results, which is in line with previous reports in the literature for comparable systems, especially for polymers with larger PLA blocks, due to the amphiphilic nature and solubility differences in various solvents [44,45,46]. The TGA traces showed two distinctive mass losses at decomposition temperatures of PLA (150–250 °C) and PEG (330–400 °C), which allowed one to roughly estimate the PEG/PLA ratio, as seen in Figures S8 and S9 and Table 1. The values for the mass fractions of PLA were converted into the mass average degree of polymerization. These values were found to be consistent with the values for the number average degree of polymerization obtained from ^1^H-NMR. The degree of polymerization obtained by TGA was in both cases to within a few % identical to the degree of polymerization determined by ^1^H-NMR (Table 1). The experimentally determined degree of polymerization also matched the nominal values of both polymers to within ~5%. 

From the panel of block copolymers, we chose PEG_114_-*b*-PLA_143_ and PEG_114_-*b*-PLA_499_ for further investigations, since these polymers can be expected to form micelles and polymersomes, respectively, considering the mass fraction of PLA (see above).

### 3.2. Encapsulation of RuPhen3 Photosensitizer into PEG_114_-b-PLA_143_ and PEG_114_-b-PLA_499_ Nanocarriers

PEG_114_-*b*-PLA_143_ and PEG_114_-*b*-PLA_499_ were used for the formation of polymersomes and micelles to encapsulate the PS via the solvent shift method, as mentioned in Section 2.2.2. 

The dispersions were characterized by DLS analysis to determine the mean hydrodynamic diameter of formed assemblies, respectively, as shown in Figures S9–S12 and Table 2. The size distributions by number obtained by DLS showed for all samples a monomodal distribution with a mean hydrodynamic diameter of close to 100 nm. Furthermore, the diameter of loaded nanocontainers was in all cases larger than non-loaded micelles and polymersomes. The values for the hydrodynamic radii are summarized in Table 2 and are shown in Figures S9–S12.

The mean hydrodynamic diameter increased upon the addition of RuPhen3 during the self-assembly for the observed polymers. However, the influence seemed to be larger on the PEG_114_-*b*-PLA_143_ assemblies. The increase in Ruphen3 amounts during formation of assemblies did not show a significant change in mean hydrodynamic diameter of the respective assembly. Repetitions of self-assembly experiments in the presence of RuPhen3 with the polymers under the same conditions showed similar results regarding size distribution and loading efficiency. 

**Figure 2:**
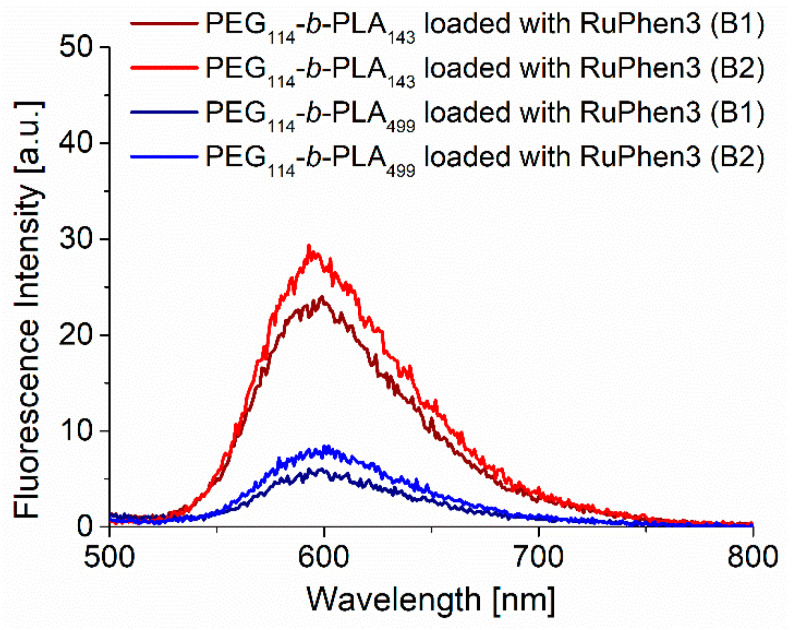
Fluorescence emission spectra of RuPhen3-loaded PEG_114_-*b*-PLA_143_ and PEG_114_-*b*-PLA_499_ assemblies formed with 2.5 (B1) and 5.0 (B2) mg/mL of RuPhen3 during the formation of micelles and polymersomes (*λ*_ex_ = 450 nm).

The concentration of RuPhen3 in the dispersions was determined by fluorescence spectroscopy, as described in Experimental Section 2.2.3, using a calibration curve (Figure S14). The fluorescence spectra (Figure 2) of RuPhen3 encapsulated in PEG_114_-*b*-PLA_143_ micelles showed a slight blue shift of around 2 nm compared with RuPhen3 encapsulated in PEG_114_-*b*-PLA_499_ vesicles and RuPhen3 in the aqueous solution (Figure S14). This blue shift might indicate different local environments of the photosensitizer inside the different assemblies. It was also shown that the fluorescence emission was higher for RuPhen3 encapsulated in PEG_114_-*b*-PLA_143_ assemblies than for PEG_114_-*b*-PLA_499_ assemblies. The doubling of the concentration of RuPhen3 during the formation of micelles and vesicles led to an increase in the RuPhen3 concentration in the final dispersion of 6% and 14%, respectively. For samples without RuPhen3, no fluorescence emission was observed.

The apparent RuPhen3 concentrations indicated a higher loading, and consequently a higher encapsulation efficiency, for the PEG_114_-*b*-PLA_143_ micelles compared with the PEG_114_-*b*-PLA_499_ polymersomes. The estimated concentrations of RuPhen3 from fluorescence spectroscopy and the determined mass concentrations of assemblies are summarized in Table 3. Due to the potential quenching of RuPhen3 or energy transfer processes, these estimates may lead to underestimating the overall RuPhen3 concentration. 

The nanocontainer dispersions showed a mass concentration of polymeric assemblies between 1.5 and 2.0 mg/mL, depending on the loading and the used polymer. The mass concentration of loaded polymersome and micelle dispersions was in all cases significantly higher, ranging from 1.8 to 1.9 mg/mL, compared with non-loaded assemblies, which exhibited values between 1.4 and 1.5 mg/mL. This mass increase was only in part due to the encapsulated RuPhen3, which would constitute a mass difference of around 0.8 mg/mL, assuming 100% of added RuPhen3 to be encapsulated. However, the mass increase was not proportional to the determined RuPhen3 concentrations of around 54.0 and 15.5 µM (B2) for PEG_114_-b-PLA_143_ and PEG_114_-b-PLA_499_ respectively, which would cause a mass difference of less than 0.1 mg/mL, but was similar for all RuPhen3-loaded samples (1.8 to 1.9 mg/mL). The higher mass concentration under identical conditions could further be attributed to the formation of larger assemblies for RuPhen3-loaded nanocarriers (B1 and B2) compared with non-loaded batches, as shown in Table 2 (Figures S9, S10 and S12). 

**Figure 3:**
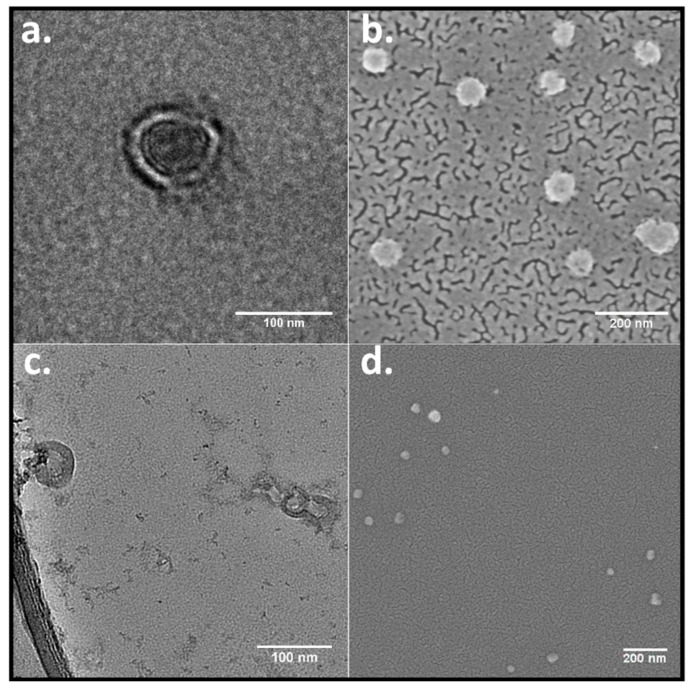
TEM images of RuPhen3-loaded (**a**) PEG_114_-*b*-PLA_143_ micelles and (**c**) PEG_114_-*b*-PLA_499_ polymersomes and FE-SEM images of (**b**) PEG_114_-*b*-PLA_143_ micelles and (**d**) PEG_114_-*b*-PLA_499_ polymersomes. The dark corona of the spherical feature as seen in Figure 3c (left side) was measured for single polymersomes and, from it, the average wall thickness was calculated, as demonstrated in Figure S13.

To confirm the morphology of the RuPhen3-loaded nanocontainers, TEM and FE-SEM microscopy images were acquired. The PEG_114_-*b*-PLA_143_ assemblies appeared in the TEM images (Figure 3a) as filled particles, consistent with block copolymer micelles, whereas PEG_114_-*b*-PLA_499_ formed assemblies with clearly discernible walls around inner circular areas that exhibited light contrast [47]. The appearance was consistent with the morphology of polymersomes. An estimate for the wall thickness afforded a mean value of 17 ± 4 nm, determined from the data of several single polymersomes, as shown in Figure 3c, by equating the difference of the outer and the inner diameter of the polymersomes. These values showed to be similar to the wall thickness for similar PEG-*b*-PLA polymersomes determined in the literature [48], stating a wall thickness of 21 ± 4 nm determined from TEM images. For this purpose, the polymersome wall thickness was measured several times at different positions for each polymersome. Consistent with all these observations, the FE-SEM images show assemblies that were predominantly spherical in shape for both polymers (Figure 3b,d). 

The overall concentration of encapsulated RuPhen3 was further used to roughly estimate the amount of encapsulated RuPhen3 per nanocarrier, considering the mass concentration and average hydrodynamic diameter. The maximum amount of RuPhen3 that could be encapsulated per nanocarrier was estimated, considering the mass concentration, total amount of encapsulated RuPhen3, average hydrodynamic diameter, and approximate wall thickness of polymersomes (17 ± 4 nm) estimated from TEM micrographs, as shown in Figure 3. The volume for micelles and shell volume for vesicles, where RuPhen3 was expected to be located due to its better solubility in hydrophobic environments, was calculated using the hydrodynamic diameter determined by DLS, assuming that all nanocarriers were spherical in shape. Furthermore, the number of micelles and vesicles per volume unit were estimated considering the mass concentration of nanocarriers in dispersion and average hydrodynamic diameter determined by DLS. The density of the polymer was assumed to be around 1.2 g/cm^3^, with respect to the density of PEG stated by the supplier and PLA polymers [49]. This estimation indicated that larger amounts of RuPhen3 were encapsulated in the internal volume of micelles (~7 × 10^3^ molecules per micelle, 35 mM) in comparison with vesicles with approximately 1 × 10^3^ molecules per polymersome (10 mM), where RuPhen3 was expected to be localized in the shell volume. These values were an order of magnitude higher than concentrations determined for similar ruthenium-based photosensitizers inside PLGA nanoparticles developed for potential use in photodynamic therapy, as described by Bœuf et al. [50].

### 3.3. Increasing the Number Concentration of Loaded Micelles and Polymersomes

The concentrations of RuPhen3 in the polymersome dispersions (15.5 μM) were potentially too low to show a sufficient reduction in bacteria in incubation and irradiation experiments, since these experiments required further dilution of the dispersion. As previously reported, RuPhen3 and comparable complexes showed to be effective down to concentrations of 10 µM [16]. Preliminary experiments with neat RuPhen3 also confirmed this and showed that RuPhen3 concentrations of <10 μM were no longer affecting the bacterial growth. Hence, the concentration of micelles and polymersomes was increased by upconcentrating the dispersions of micelles and vesicles. To this end, the dispersions were centrifuged. The supernatant volume was reduced by the removal of the medium, and the pellet of sedimented nanocarriers was redispersed in a reduced volume of dispersant. This led to an increase in the mass concentration and concentration of encapsulated PS by a factor of 10.

To obtain sufficient volumes of loaded micelle and polymersome dispersions for different incubation and irradiation experiments, the batch size was increased fourfold, as described in Experimental Section 2.2.2 (B3). The increase in batch size led to a reduction in concentration of encapsulated RuPhen3 of around 60–70% from 54.0 ± 1.8 μM to 16.0 ± 0.2 μM for the micelles. Furthermore, a broadening of the size distribution was observed, while the mean hydrodynamic diameter was nearly constant. By contrast, the encapsulation efficiency of PS and size distribution of polymersomes for the PEG_114_-*b*-PLA_499_ polymer were not affected significantly by the upscaling.

The number concentration of micelles or polymersomes, and thus the concentration of encapsulated PS in the final dispersions, was increased 10-fold via reducing the mass of dispersant (water) proportionally. The concentrations of PS and average hydrodynamic diameters and mass concentrations of the micelles and polymersomes are summarized in Table 4. The size distribution of micelles and polymersomes was not markedly affected upon removal of the dispersant volume.

To prove that repetitive centrifugation did not affect the size distribution drastically, DLS was applied, and the mass concentration was determined after 10 centrifugation, supernatant exchange, and redispersion cycles. The mean hydrodynamic diameter did not change in this process, as shown in Table 5. The mass concentration of PEG_114_-*b*-PLA_499_ assemblies was measured before and after 3-, 5-, 7-, and 10-times centrifugation, solvent exchange, and redispersion, and remained constant at 1.9 ± 0.1 mg/mL.

### 3.4. Enzymatic Release of PS from Polymeric Micelles and Polymersomes

The enzymatically triggered release of PS was achieved using the model enzyme proteinase K, which was expressed among others by the fungus *Tritirachium album*. This type of proteinase can hydrolyze peptide and ester bonds, such as the bonds present in PLA polymers. In line with earlier reports [33,34], proteinase K was expected to destabilize micelles and polymersomes containing PLA polymers, resulting in the release of encapsulated RuPhen3. The enzymatic attack should enable the released photosensitizer to diffuse to the targeted bacteria, leading to more efficient eradication of the bacteria by aPDT.

**Figure 4:**
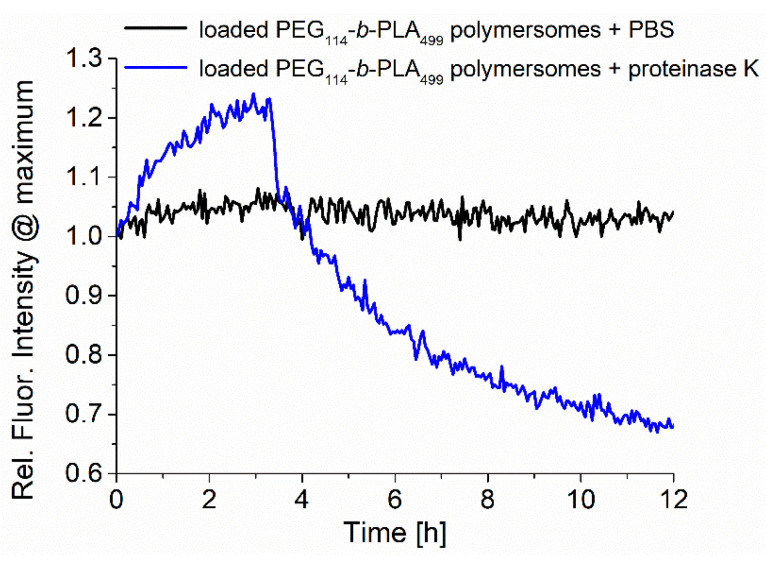
Normalized fluorescence emission of RuPhen3-loaded PEG_114_-*b*-PLA_499_ polymersomes in the presence of the enzyme proteinase K and PBS buffer (blank) (recorded at *λ*_em_ = 600 nm, *λ*_ex_ = 450 nm).

The samples were analyzed during enzymatic release by recording the fluorescence emission, as shown in Figure 4. The normalized fluorescence spectra of RuPhen3-loaded PEG_114_-*b*-PLA_499_ polymersomes after the addition of proteinase K in PBS (5.0 mg/mL) and neat PBS as a control were recorded for 12 h, and the maximum intensity at ~600 nm was plotted as a function time. While the fluorescence of RuPhen3 in the absence of proteinase K remained constant over time with few minor fluctuations, the presence of proteinase K led to a significant initial increase, followed by a rapid drop in fluorescence emission intensity. After 12 h, sedimentation of the polymer and PS was observed inside the cuvette, implying that the degradation of polymersomes due to the enzyme cleaving of PLA repetition units under the formation of lactic acid caused the polymersomes to disintegrate. When the sediments were redispersed, the fluorescence emission reached a level comparable to the maximum fluorescence emission observed after approximately 3 h after the addition of proteinase K. The increasing fluorescence could be caused by the release of the photosensitizer from the polymersomes and reduced quenching, caused initially by the high local concentration of PS inside the polymersomes. 

**Figure 5:**
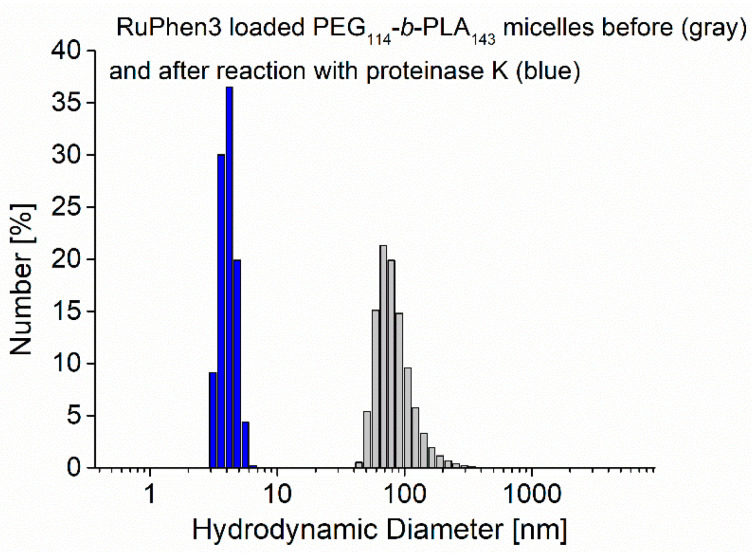
Size distribution by number (DLS) of RuPhen3-loaded PEG_114_-*b*-PLA_143_ micelles before (gray) and after 24 h incubation with proteinase K (blue).

Additionally, the size distribution of the assemblies was analyzed with DLS before and 24 h after enzymatic degradation. The size distributions showed a strong shift from an initial mean hydrodynamic diameter of 73 ± 22 nm to lower values of the hydrodynamic diameter of 4 ± 1 nm, as shown in Figure 5, which was consistent with comparable systems reported in the literature [33,34].

**Figure 6:**
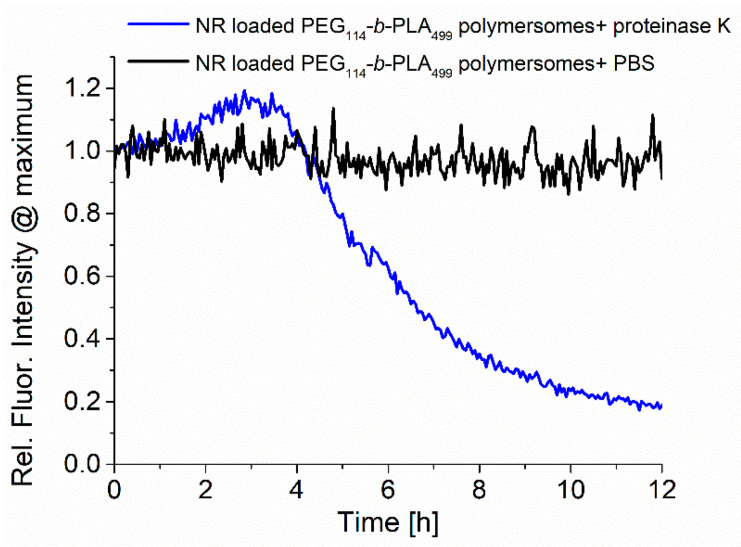
Fluorescence emission of Nile Red-loaded PEG_114_-*b*-PLA_499_ polymersomes in the presence of the enzyme proteinase K (5 mg/mL) and PBS buffer (blank) (recorded at *λ*_em_ = 600 nm, *λ*_ex_ = 450 nm).

A similar behaviour could be observed for the enzymatic degradation of PEG_114_-*b*-PLA_499_ polymersomes loaded with the model dye Nile Red, as shown in Figure 6. The change in fluorescence emission around 620 nm showed a comparable increase within the first 3 h, followed by a rapid decrease due to the precipitation of insoluble Nile Red and polymer upon degradation of the polymersomes, as reported in the literature [33,34].

### 3.5. Detection of Singlet Oxygen Production

**Figure 7:**
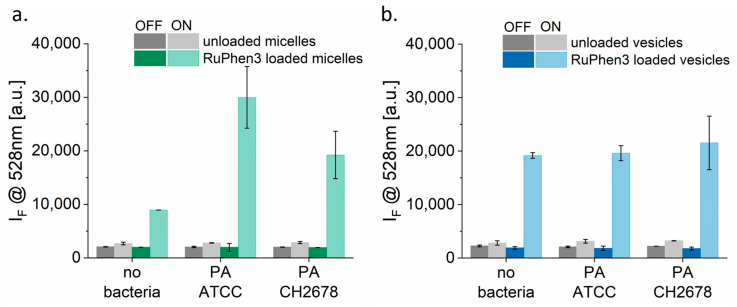
SOSG probe signal for detecting singlet oxygen production by (**a**) unloaded and RuPhen3-loaded micelles in PEG_114_-*b*-PLA_143_; (**b**) unloaded and RuPhen3-loaded vesicles in PEG_114_-*b*-PLA_499_ in the presence of and without PA ATCC and PA CH2678. (For loaded micelles, final (RuPhen3) in solution = 82.55 ± 7.70 µM; for loaded vesicles, final (RuPhen3) in solution = 93.45 ± 6.55 µM). The incubation time of the samples with the bacteria suspensions was 15 min prior to the addition of the SOSG solution. The samples were irradiated at *λ* = 455 nm for 30 min (ON) or were incubated in the dark for 30 min (OFF).

The well-known SOSG probe was used to detect the singlet oxygen production in both irradiated and non-irradiated samples [51]. The fluorescence intensity of the probe signal after a certain irradiation time was measured. Fluorescence was only observed in the presence of a photosensitizer that produced ^1^O_2_ species, which were then detected by the probe [51]. Figure 7 shows the SOSG probe signal of unloaded and RuPhen3-loaded micelles and vesicles incubated with and without bacterial strains PA ATCC and PA CH2678 in 0.9% saline. The concentrations of the RuPhen3-loaded micelles (165.1 ± 15.4 µM) and vesicles (186.9 ± 13.1 µM) given in Table 4 were used for the experiments. For both the SOSG testing and aPDT experiments, the loaded micelles and vesicles were mixed in 1:1 ratio with the studied different mediums to have their final concentrations to be ~half of the undiluted loaded micelles and vesicles. 

For unloaded micelles (Figure 7a) and vesicles (Figure 7b), a slight increase in probe signal was observed after irradiation. This increase corresponded to the signal originating from the SOSG probe itself, which underwent photochemistry to a fluorescent product through a different mechanism [51]. The non-irradiated RuPhen3-loaded micelles and vesicles did not show any significant change in fluorescence intensity. 

By contrast, a strong increase in fluorescence intensity was observed for irradiated RuPhen3-loaded micelles and vesicles. For loaded micelles, a significantly higher probe signal was observed for samples incubated with bacteria compared with samples incubated in 0.9% NaCl (i.e., without bacteria). The fluorescence of the probe also differed among the two bacterial strains. However, the probe signal did not vary for loaded vesicles measured with and without bacteria in sterile water or among the two strains. These observations indicate that the bacteria may affect the micelles differently compared with the polymersomes. 

For the controls, the probe itself (diluted in tris buffer, pH 7.5) and both bacterial strains (in 0.9% saline) were also irradiated in the presence of SOSG (Figure S18a). After irradiation, a small increase in fluorescence intensity was observed in all cases that corresponded to the mentioned side reaction of SOSG probe during irradiation [51]. In addition, a significant increase in probe signal was observed after the irradiation, when neat RuPhen3 was incubated without and in the presence of the bacterial strains, at the same concentrations as both the micelles and vesicles (Figure S18b). A higher probe signal was observed when RuPhen3 was irradiated and incubated in bacterial suspensions compared with the bacteria-free solution. As the intensity was lower in the absence of any polymer, this indicated that the probe may partition into the nanocontainers’ PEG corona, which in turn may alter the reacted probes’ quantum yield.

### 3.6. aPDT Effect on Planktonic Bacteria

**Figure 8:**
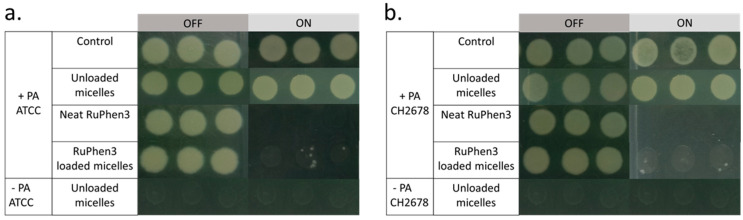
Exemplary photographs of spotted bacterial colonies (after growth for 18 h) of (**a**) PA ATCC and (**b**) PA CH2678 without and with irradiation at *λ* = 455 nm of bare RuPhen3 and RuPhen3-loaded micelles (average diameter 89 ± 44 nm, mass concentration in suspension 17.3 ± 1.0 mg/mL) in PEG_114_-*b*-PLA_143_. (Final neat (RuPhen3) = 82.55 µM, final (RuPhen3) in loaded micelle solution = 82.55 ± 7.70 µM.) The samples were irradiated for 30 min (ON) or incubated in the dark for 30 min (OFF).

To investigate the aPDT effect of the RuPhen3-loaded micelles and vesicles on PA ATCC and PA CH2678, the nanocarriers were added to the respective bacterial suspensions and were subjected to irradiation following incubation for 15 min. Afterwards, serial dilutions of each sample were performed and three times 5 µL of each dilution was plated to count the CFU/mL for both the irradiated and non-irradiated samples. For comparison, the aPDT effect of neat RuPhen3 was also investigated. The spotting results of the bacteria colonies of RuPhen3-loaded micelles for non-irradiated and irradiated samples incubated with both bacteria strains are shown in Figure 8. No effect of irradiation was observed for the bacteria controls and unloaded micelles incubated with bacteria suspension. After irradiation, neat RuPhen3 showed no visible colonies, while, for RuPhen3-loaded micelles, very few colonies were visible. The faint background color of RuPhen3-loaded micelles after irradiation was very similar to that of spotted unloaded micelles. No dark toxicity was obvious.

**Figure 9:**
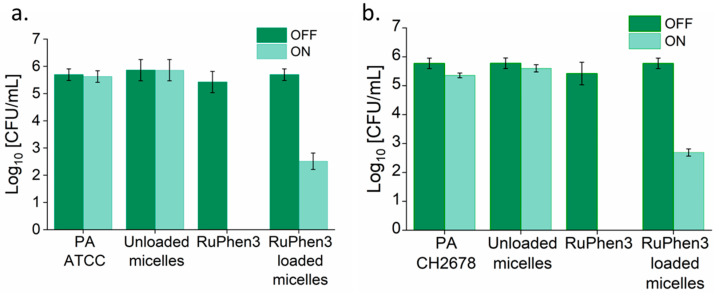
Bar plot of log_10_ CFU/mL calculated by the plate count method for (**a**) PA ATCC and (**b**) PA CH2678 incubated with neat RuPhen3 and RuPhen3-loaded micelles for irradiated and non-irradiated samples (compare Figure 8). (Final neat (RuPhen3) = 82.55 µM, final (RuPhen3) in loaded micelle solution = 82.55 ± 7.70 µM.) The samples were irradiated for 30 min (ON) or incubated in the dark for 30 min (OFF). Absence of bar: no countable colonies. The error bars correspond to the standard deviation of technical replicates (*n* = 3).

Analysis of the serial dilutions afforded the log_10_ CFU/mL values of the RuPhen3-loaded micelles incubated with each bacterial suspension (Figure 9).

The non-irradiated samples showed similar CFUs compared to the corresponding bacteria controls. Neat RuPhen3 showed no countable bacteria after the irradiation, while the RuPhen3-loaded micelles showed a > 3 log_10_ CFU/mL for each bacterial strain after the irradiation.

**Figure 10:**
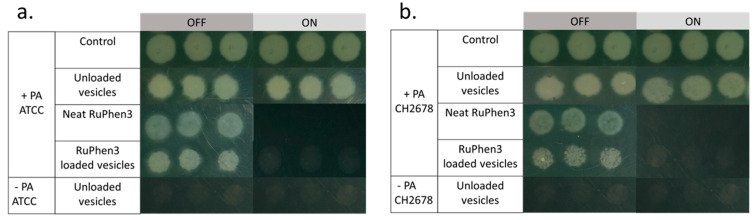
Exemplary photographs of spotted of bacterial colonies (after growth for 18 h) of (**a**) PA ATCC and (**b**) PA CH2678 without and with irradiation at *λ* = 455 nm of bare RuPhen3 and RuPhen3-loaded vesicles (average diameter 74 ± 29 nm, mass concentration in suspension 17.9 ± 0.6 mg/mL) in PEG_114_-*b*-PLA_499_. (Final neat (RuPhen3) = 93.45 µM, final (RuPhen3) in loaded vesicle solution = 93.45 ± 6.55 µM.) The samples were irradiated for 30 min (ON) or incubated in the dark for 30 min (OFF).

Similarly, the aPDT effect was investigated for RuPhen3-loaded vesicles. Figure 10 shows the spotting results of bacterial colonies for non-irradiated and irradiated samples. After the irradiation, there were no colonies observed for both the neat RuPhen3 and RuPhen3-loaded vesicles. Unloaded vesicles were also plated and resulted in very faint but discernible spots that were attributed to the scattering of light due to the dried vesicles. Hence, the very faint spots observed in Figure 10 for irradiated vesicle samples were attributed to the vesicles themselves.

**Figure 11:**
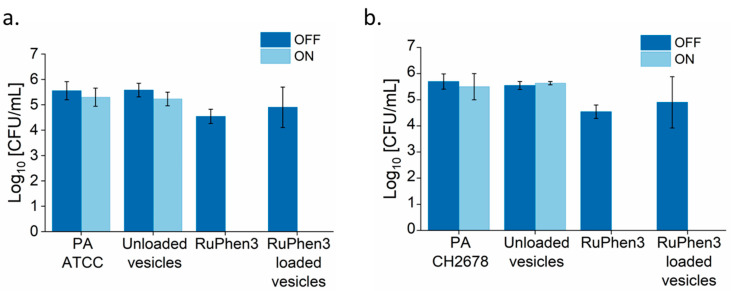
Bar plot of log_10_ CFU/mL calculated by the plate count method for (**a**) PA ATCC and (**b**) PA CH2678 incubated with neat RuPhen3 and RuPhen3-loaded vesicles for irradiated and non-irradiated samples (compare Figure 10). (Final neat (RuPhen3) = 93.45 µM, final (RuPhen3) in loaded vesicle solution = 93.45 ± 6.55 µM.) The samples were irradiated for 30 min (ON) or incubated in the dark for 30 min (OFF). Absence of bar: no countable colonies. The error bars correspond to the standard deviation of technical replicates (*n* = 3).

The dilutions were analyzed and the log_10_ CFU/mL values of the RuPhen3-loaded vesicles were then calculated (Figure 11). No changes in CFU/mL were observed for the non-irradiated samples. Both for neat RuPhen3 and RuPhen3-loaded vesicles, no countable CFUs were observed after the irradiation.

The LRF values were then calculated by comparing each sample to respective non-irradiated bacterial suspensions. While calculating the LRF values, the LOD values of the biological experiments (67 CFU/mL) were also explicitly considered, which meant that the LRF values reported here were lower bounds. Table 6 summarizes the resulting LRFs of the RuPhen3-loaded micelles and vesicles. For the non-irradiated RuPhen3-loaded micelles and vesicles, an LRF < 0.8 was observed. For irradiated samples, LRF values > 3 were observed for loaded micelles, while LRF values > 4.7 were observed for RuPhen3-loaded vesicles. The RuPhen3-loaded vesicle formulations thus showed a much higher bacteria inactivation capacity compared with the RuPhen3-loaded micelles.

As controls, the LRF values for non-irradiated and irradiated neat RuPhen3 and unloaded micelles and vesicles incubated with respective bacterial suspensions are shown in Table 7. For non-irradiated and irradiated unloaded micelles and vesicles, < 0.5 LRF was observed. The non-irradiated neat RuPhen3 showed ~2 LRF, while the irradiated one showed LRF values of >4. 

## 4. Discussion

PEG_114_-*b*-PLA block copolymers with different PLA block lengths were synthesized and characterized using ^1^H-NMR and TGA (Figures S1–S8 and Table S1). Micelles and vesicles were formed via the solvent shift method with PEG_114_-*b*-PLA_143_ and PEG_114_-*b*-PLA_499_, respectively, and yielded nanocarriers with average hydrodynamic diameters below 100 nm. The assignment to micelles and vesicles was carried out for RuPhen3-loaded assemblies on the basis of TEM and FE-SEM data (Figure 3), in accordance with the literature of unloaded assemblies [47]. The TEM images showed spherical micelles for the PEG_114_-*b*-PLA_143_ polymer assemblies, while, for the PEG_114_-*b*-PLA_499_ polymer, spherical but hollow polymersome-like structures were observed. This influence of the block length of PLA on the morphology of the assemblies is well known in the literature and has attributed to a change in packing parameters, which depend on the length and volume of the hydrophobic chain and the optimal area of the hydrophilic head group for amphiphilic molecules [52]. RuPhen3 could be successfully encapsulated into micelles and polymers with total concentrations up to 54.0 μM that were already sufficient for antimicrobial effects upon irradiation (Table 7). The increase in RuPhen3 amounts did not lead to a proportional increase in encapsulated RuPhen3 in the dispersions but only a minor increase upon reduction of encapsulation efficiency. However, the batch size could be increased, and assemblies upconcentrated in dispersion by increasing the mass concentration and number density upon centrifugation and reduction of dispersant to further increase the overall RuPhen3 concentration.

Via fluorescence spectroscopy, it was confirmed that the PEG_114_-*b*-PLA_143_ micelles and PEG_114_-*b*-PLA_499_ polymersomes were affected by the presence of the enzyme proteinase K in the same way that has previously been reported for PEG-*b*-PLA block copolymers (Figure 4 and Figure 6) [33,34]. The enzyme-triggered PLA degradation facilitated the release of the encapsulated RuPhen3. The release coincided with an initial increase in fluorescence, attributed to a dilution of the PS and hence dequenching upon release (Figure 4). The subsequent rapid decrease in fluorescence intensity was attributed to the precipitation of polymer together with the PS in the enzyme buffer, which was qualitatively similar for the light-off reporter dye Nile Red (Figure 6). In comparison to other enzyme-triggered systems used for aPDT, which showed either enzyme-triggered release of PS from PEG-*b*-PCL [53] or of signaling dyes to detect bacterial enzymes and bacteria using PEG-*b*-PLA or PEG-*b*-PCL polymers [33,34], this current approach system could be further developed for improved simultaneous detection and treatment of bacterial infections towards a theranostic device, since it combines the properties of an enzyme-sensing fluorescent probe with a high aPDT potential, as shown for *Pseudomonas aeruginosa*.

The singlet oxygen species produced by RuPhen3 from loaded nanocarriers were detected by measuring the fluorescence intensity of the SOSG probe. RuPhen3 complexes absorbed photons and were excited from the ground state to a short-lived excited state, which could then be converted into a longer-lived triplet excited state via inter-system crossing. From this state, reactive oxygen species could be produced by either electron transfer or energy transfer, which occurred simultaneously [35]. These singlet oxygen species then reacted with the anthracene unit of the SOSG probe (non-fluorescent) to form its endoperoxide form (Figure S19), which showed green fluorescence with an emission maximum at 525 nm when excited at 504 nm. The SOSG probe itself was observed to show an increase in fluorescence intensity, even in the absence of any photosensitizer. This observation has also been reported in the literature and has been attributed to different conversion mechanisms of the SOSG probe that contributed to the final probe signal [51,54,55]. In case of unloaded micelles and vesicles, no significant change in the SOSG probe signal was observed for irradiated and non-irradiated samples. For RuPhen3-loaded micelles and vesicles, the SOSG probe signal increased significantly after the irradiation, indicating ^1^O_2_ production. 

Moreover, for irradiated RuPhen3-loaded micelles, the SOSG probe signal increased when incubated with bacterial suspensions (Figure 7a). By contrast, for RuPhen3-loaded vesicles, no significant change in probe signal was observed for samples incubated with or without bacterial suspensions (Figure 7b). The detection of ^1^O_2_ species depended on various factors, including the concentration of the PS and the local oxygen concentration. In addition, the lifetime of ^1^O_2_ and its diffusion rate to reach the probe were important, which both changed according to the surrounding media (viscosity, presence of other reactants, etc.). 

The aPDT effect of RuPhen3-loaded micelles and vesicles against *Pseudomonas aeruginosa*, both lab strain and clinical CF isolate, was very pronounced according to the results summarized in Table 6. For RuPhen3-loaded micelles, the agar spotting method revealed that there were only a few countable colonies in the non-diluted sample (Figure 8), equating to an at least 3-log reduction of the bacteria after irradiation (see LRF, Table 7). This means that a bactericidal effect was observed, which was defined as the inactivation of 99.9% of the initially viable (here, colony forming) bacteria. For RuPhen3-loaded vesicles, no visible colonies could be identified after irradiation for either bacterial strain (Figure 10), referring to an LFR > 4.7 when calculating the reduction of CFU/mL in each individual experiment compared with the control (Table 6). So, higher PDI efficiency was observed for RuPhen3-loaded vesicles in the formulations used compared with RuPhen3-loaded micelles and even with neat RuPhen3 (Table 7). The mechanisms for this improvement via the nanocarrier require further investigation. One may hypothesize that, apart from stabilizing the PS itself, the nanocarriers may affect the interaction of RuPhen3 with the bacterial cell wall, which depends both on the bacterial pathogen and its wall structure (Gram-positive or Gram-negative) and the surrounding media [27,36].

When comparing the SOSG probe signal intensities of RuPhen3-loaded micelles and vesicles incubated with bacterial suspensions, higher SOSG signal intensities were observed for loaded micelles compared with loaded vesicles after irradiation, while the aPDT results showed the opposite trend. Higher LRFs were observed for loaded vesicles compared with micelles. Therefore, it was not a priori valid to directly correlate the SOSG probe signal with the aPDT efficiency of the systems. Several other factors are required to be considered, such as the interaction of bacteria with the nanocarriers, the availability of the produced singlet oxygen species to reach the bacteria [35], and various effects that reduce the quantum yields of the reporter probe.

As a final goal with high medical impact, aPDT should achieve a complete eradication of bacteria in order to avoid the recolonization of infected tissue, wound dressings, etc. Although RuPhen3-loaded micelles also resulted in a clear bactericidal effect with an LRF > 3, only the RuPhen3-loaded vesicle formulations afforded a complete killing in this study. This effect even exceeded the photodynamic inactivation (PDI) efficiency of the neat RuPhen3 (Table 6 and Table 7). Hence, the encapsulation of RuPhen3 in vesicles may enhance the PDI efficiency for planktonic *Pseudomonas aeruginosa*. A similar effect was observed by Mirzahosseinipour et al., who compared the aPDT effect of curcumin and curcumin encapsulated in silica nanoparticles [56]. They observed a further reduction in the CFU/mL of planktonic bacteria for curcumin-encapsulated silica nanoparticles compared with neat curcumin. The curcumin silica nanoparticles were shown to improve the activity of curcumin due to good uptake and its controlled release.

The RuPhen3-loaded PEG_114_-*b*-PLA_143_ micelles showed similar aPDT efficiency of 99.9% inactivation (3-log reduction) compared with the comparable system of lipase-sensitive PEG-*b*-PCL micelles reported by Guo et al. [53]. However, the RuPhen3-loaded PEG_114_-*b*-PLA_499_ polymersomes showed an antimicrobial effect with a >4.7-log reduction, which was comparable, but they required less light intensity compared with reports about similar polymeric nanocarrier systems. Microparticles irradiated with red light power of 9.28 mW/cm^2^ for 30 min or other types of inorganic nanoparticles, e.g., gallium-substituted silver nanoparticles irradiated with nearly 50 times higher light intensity but only 30s irradiation of blue light as used in this study, were previously reported [57,58].

The slightly higher log-reduction factor observed for polymersomes with encapsulated RuPhen3 compared with neat RuPhen3 might be explained by the improved solubility of RuPhen3 upon encapsulation and thus a higher local concentration, which would be enhanced if an enzymatically triggered release of RuPhen3 from the polymersomes occurred. In the absence of bacteria, the release was shown here to be triggered by a model enzyme, as shown in Figure 4 and Figure 6. It can also be envisioned that high local concentrations of RuPhen3 in polymersomes result in higher concentrations of ^1^O_2_ upon irradiation if the polymersomes reside close to the bacteria.

The herewith-reported systems could be further improved by the use of more efficient ruthenium-based photosensitizers, as reported in the recent literature [16,35,59,60], or photosensitizers that have been shown to be efficient against various bacteria, e.g., methylene blue or rose bengal. Also, PS molecules, which are excited at longer wavelengths, could be used for encapsulation, since these PSs might be easier to excite within biological media or tissues due to the more pronounced penetration depths of light.

## 5. Conclusions

In this study, it was shown that different PEG_114_-*b*-PLA polymers, synthesized by ring-opening polymerization and characterized regarding composition and degree of polymerization, could be used to form polymeric nanocarriers (micelles and polymersomes) to encapsulate the photosensitizer RuPhen3. Preliminary results showed the highest encapsulation efficiency for PEG_114_-*b*-PLA_143_ and PEG_114_-*b*-PLA_499_ polymers. It could be proven that the morphology of the nanocarrier depended on the degree of polymerization of PLA and the experimental conditions, leading to the formation of micelles for the PEG_114_-*b*-PLA_143_ and polymersomes for the PEG_114_-*b*-PLA_499._ The loading efficiency of PS in polymeric micelles and polymersomes strongly depended on the used polymer/morphology of assembly. Loaded micelles and polymersomes with mean hydrodynamic diameters below 100 nm and overall RuPhen3 concentrations of up to 54.0 ± 1.8 μM and 18.7 ± 1.0 μM, respectively, were formed and upconcentrated. A *Pseudomonas aeruginosa* lab strain (PA ATCC 19660) and a relevant clinical CF-Isolate (CH2678) were eradicated in vitro by aPDT with blue light during 30 min of irradiation time. Compared with the micelles, the PS-loaded vesicles showed 10 times enhanced activity, with a strong photodynamic inactivation effect of at least a 4.7 log reduction against both strains. The results show the feasibility of the approach to enhance locally and on demand the aPDT effect and lay the foundation for the targeted eradication of *Pseudomonas aeruginosa* using aPDT in various application areas.

## Figures and Tables

**Table 1:** Values of the degree of polymerization of the PLA blocks of the synthesized PEG_114_-b-PLA_X_ polymers using ^1^H-NMR spectroscopy and TGA data.

Polymers and Nominal Degree of Polymerization	Number Average Degree of Polymerization	Approximate Mass Average Degree of Polymerization
	(^1^H-NMR Spectroscopy)	(TGA)
*PEG*_114_ *-b-PLA*_150_	143	139
*PEG*_114_ *-b-PLA*_500_	499	491

**Table 2:** Size (mean hydrodynamic diameter) of PEG_114_-b-PLA_X_ assemblies formed in the presence of and without RuPhen3 photosensitizer.

Polymer	Mean Hydrodynamic Diameter (nm) from Size Distributions by Number (DLS)		
	Batch1	Batch2	Non-Loaded
*PEG*_114_ *-b-PLA*_143_	89 ± 35	86 ± 34	66 ± 28
*PEG*_114_ *-b-PLA*_499_	75 ± 22	73 ± 22	70 ± 22

**Table 3:** Concentration of RuPhen3 in nanocontainer dispersions formed from different PEG_114_-*b*-PLA_X_ polymers estimated by fluorescence emission and mass concentration of PEG_114_-*b*-PLA_X_ nanocontainers in dispersion measured by mass loss upon evaporation of solvent. The results obtained using an analytical balance were cross-confirmed by TGA measurements. The corresponding fluorescence spectra for different concentrations of RuPhen3 are shown in Figure S14.

Polymer Assembly	Concentration of RuPhen3 (μM)		
	Batch1	Batch2	
*PEG*_114_*-b-PLA*_143_ *micelles*	47.2 ± 3.2	54.0 ± 1.8	
*PEG*_114_*-b-PLA*_499_ *vesicles*	14.6 ± 0.2	15.5 ± 0.2	
	**Mass concentration (mg/mL)**		
	**Batch1**	**Batch2**	**Non-loaded**
*PEG*_114_*-b-PLA*_143_ *micelles*	1.8 ± 0.1	1.8 ± 0.1	1.5 ± 0.1
*PEG*_114_*-b-PLA*_499_ *vesicles*	1.8 ± 0.1	1.9 ± 0.1	1.4 ± 0.1

**Table 4:** Mean hydrodynamic diameters (from DLS) of PEG_114_-*b*-PLA_143_ and PEG_114_-*b*-PLA_499_ assemblies, estimated RuPhen3 concentration, and mass concentration of assemblies before and after upconcentration for Batch3.

Polymer	Mean Diameter (nm)	RuPhen 3 Conc. (μM)	Mass Conc. (mg/mL)	RuPhen 3 Conc. (μM) after	Mass Conc. (mg/mL) after
*PEG*_114_*-b-PLA*_143_ *micelles*	89 ± 44	16.0 ± 0.2	1.7 ± 0.1	165.1 ± 15.4	17.3 ± 1.0
*PEG*_114_*-b-PLA*_499_ *vesicles*	74 ± 29	18.7 ± 1.0	1.8 ± 0.1	186.9 ± 13.1	17.9 ± 0.6

**Table 5:** Mean hydrodynamic diameters of PEG_114_-*b*-PLA_143_ and PEG_114_-*b*-PLA_499_ assemblies before and after 10-times centrifugation at 6000 rpm for 20 min and redispersion.

Polymer	Mean Hydrodynamic Diameter (nm)	
	Batch2	Batch2 10× Centrifuged and Redispersed
*PEG*_114_*-b-PLA*_143_ *micelles*	86 ± 34	88 ± 36
*PEG*_114_*-b-PLA*_499_ *vesicles*	73 ± 22	76 ± 30

**Table 6:** LRF values of the non-irradiated (OFF) and irradiated (ON) RuPhen3-loaded micelles and vesicles. The LRF was calculated by comparing the data to the non-irradiated bacteria controls (*n* = 3).

	RuPhen3-Loaded			
	Micelles		Vesicles	
	OFF	ON	OFF	ON
PA ATCC	−0.01 ± 0.03	3.15 ± 0.23	0.65 ± 0.22	4.75 ± 0.23
PA CH2678	0.07 ± 0.02	3.40 ± 0.51	0.81 ± 0.17	4.90 ± 0.17

**Table 7:** LRF calculations of the neat RuPhen3 after light irradiation. LRF is calculated by comparing the respective non-irradiated bacteria controls (*n* = 3 for unloaded micelles and vesicles, *n* = 6 for neat RuPhen3).

	Neat RuPhen3		Unloaded Micelles		Unloaded Vesicles	
	OFF	ON	OFF	ON	OFF	ON
PA ATCC	1.98 ± 0.22	4.28 ± 0.66	−0.25 ± 0.04	−0.24 ± 0.01	1.00 ± 0.23	1.32 ± 0.24
PA CH2678	2.14 ± 0.18	4.44 ± 0.65	0.01 ± 0.02	0.17 ± 0.03	1.18 ± 0.18	1.08 ± 0.16

## Data Availability

The data presented in this study are available in this article (and supplementary material) or on request from the corresponding authors.

## Supplementary Materials

The following supporting information can be downloaded at: https://www.mdpi.com/article/10.3390/pharmaceutics15122683/s1, Figure S1: ^1^H-NMR spectrum of PEG_114_-*b*-PLA_143_ (solvent CDCl_3_); Figure S2: ^1^H-NMR spectrum of PEG_114_-*b*-PLA_499_ (solvent CDCl_3_); Figure S3: ^1^H-NMR Spectrum of PEG_114_-*b*-PLA_48_ solvent (DMSO-*d*_5_); Figure S4: ^1^H-NMR spectrum of PEG_114_-*b*-PLA_112_ (solvent CDCl_3_); Figure S5: ^1^H-NMR spectrum of PEG_114_-*b*-PLA_207_ (solvent CDCl_3_); Figure S6: ^1^H-NMR Spectrum of PEG_114_-*b*-PLA_301_ (solvent CDCl_3_); Figure S7: ^1^H-NMR Spectrum of PEG_114_-*b*-PLA_408_ (solvent CDCl_3_); Figure S8: TGA traces of the synthesized PEG_114_-*b*-PLAX polymers with a heating rate of 5 °C/min from 200 to 450 °C under N_2_ atmosphere; Figure S9: Size Distributions (DLS) of PEG_114_-*b*-PLA_499_ polymersomes in water (Batches 1–3); Figure S10: Size Distributions (by DLS) of PEG_114_-*b*-PLA_143_ micelles in water (Batches 1–3); Figure S11: Size Distributions (by DLS) of PEG_114_-*b*-PLA_143_ micelles and PEG_114_-*b*-PLA_499_ polymersomes after 10× centrifugation and solvent exchange in water (Batch 2); Figure S12: Size Distributions (by DLS) of non-loaded PEG_114_-*b*-PLA_143_ micelles and PEG_114_-*b*-PLA_499_ polymersomes in water; Figure S13: TEM images of RuPhen3-loaded PEG_114_-*b*-PLA_499_ polymersomes as shown in Figure 3 with indicated wall thickness (compare arrow); Figure S14: Fluorescence intensity at the maximum emission for different RuPhen3 concentrations in aqueous solution as a function of concentration (left) and fluorescence emission spectra of RuPhen3 in aqueous solution for concentrations between 250 nM and 50 μM (right) excited at 450 nm; Figure S15: Fluorescence emission spectra of RuPhen3 loaded PEG_114_-*b*-PLA_143_ micelles and PEG_114_-*b*-PLA_499_ polymersomes in aqueous solution (Batch3) for upscaled batches excited at 450 nm; Figure S16: Fluorescence emission spectra of RuPhen3 (excited at 450 nm) encapsulated into PEG_114_-*b*-PLA_499_ polymersomes over minimum 12 h in presence of proteinase K (5 mg/mL) in PBS (left) and without addition of enzyme in PBS (right); Figure S17: Fluorescence emission spectra of Nile Red (NR) (excited at 530 nm) encapsulated into PEG_114_-*b*-PLA_499_ polymersomes over minimum 12 h in presence of proteinase K (5 mg/mL) in PBS (left) and without addition of enzyme in PBS (right); Figure S18: SOSG probe signal for detecting singlet oxygen production by (a) SOSG probe and the used bacterial strains PA ATCC and PA CH2678, (b) neat RuPhen3 incubated with and without bacteria strains; Figure S19: The conversion of the SOSG probe into its endoperoxide form in the presence of singlet oxygen; Table S1: Values of degree of polymerization of the PLA block of the synthesized PEG_114_-*b*-PLAX polymers using ^1^H-NMR and by weight determined with thermogravimetric analysis (TGA).
