# Supplementary material for: Photodynamic Eradication of Pseudomonas aeruginosa with Ru-Photosensitizers Encapsulated in Enzyme Degradable Nanocarriers
Source: Pharmaceutics. 2023 Nov 27;15(12):2683. doi: 10.3390/pharmaceutics15122683 (PMC10747122; doi:10.3390/pharmaceutics15122683)
Supplement: Supplementary file 1 [file pharmaceutics-15-02683-s001.zip › pharmaceutics-2701745-supplementary.pdf]

Photodynamic Eradication of *Pseudomonas aeruginosa* with Ru-Photosensitizers Released from Enzyme Degradable Nanocarriers

Kawaljit Kaur[1], Max Müller[1], Mareike Müller,* and Holger Schönherr*

Physical Chemistry I & Research Center of Micro- and Nanochemistry and (Bio)Technology (***Cμ***), Department of Chemistry and Biology, School of Science and Technology, University of Siegen, 57076 Siegen, Germany

[1]These two authors contributed equally to this work.

Supporting Information

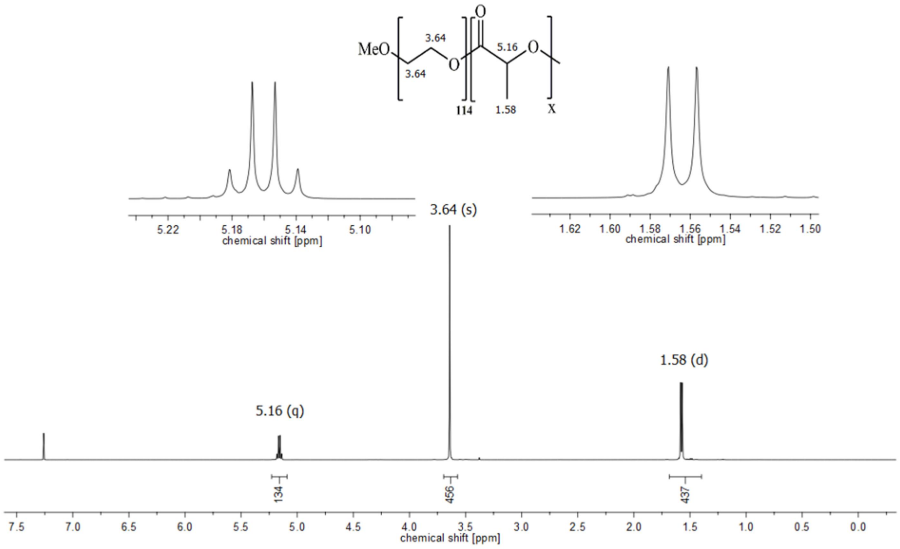

Figure S1: ^{1}H-NMR spectrum of PEG_{114}-*b*-PLA_{143} (solvent $CDCl_3$).

^{1}H-NMR (500 MHz, $CDCl_3$):δ (ppm) = 7.26 (s, $CDCl_3$): 5.16 (q, ^3J =J 7.1 Hz, 134H, $CH_3CH(C{=}O)O$ (PLA)), 3.64 (s, 456H, OCH_2CH_2O (PEG)), 3.36 (s, 3H, OCH_3) (PEG-OMe)), 1.58 (d, ^3J =7.2 Hz, 437H, $CH_3CH(C{=}O)O$ (PLA)) , 1.48 (d, ^3J =7.2 Hz, 6H , $CH_3CH(C{=}O)O$ (monomer)).

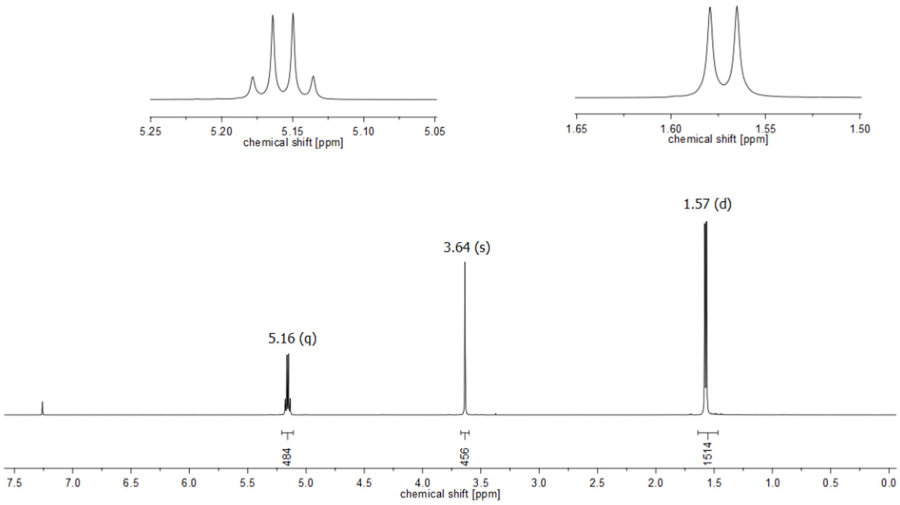

Figure S2: ^{1}H-NMR spectrum of PEG_{114}-*b*-PLA_{499} (solvent $CDCl_3$)..

^{1}H-NMR (500 MHz, $CDCl_3$):δ (ppm) = 7.26 (s, $CDCl_3$): 5.16 (q, ^{3}J =J 7.1 Hz, 484H, $CH_3CH(C{=}O)O$ (PLA)), 3.64 (s, 456H, OCH_2CH_2O (PEG)), 3.36 (s, 3H, OCH_3) (PEG-OMe)), 1.57 (d, ^{3}J =7.2 Hz, 1514H, $CH_3CH(C{=}O)O$ (PLA)).

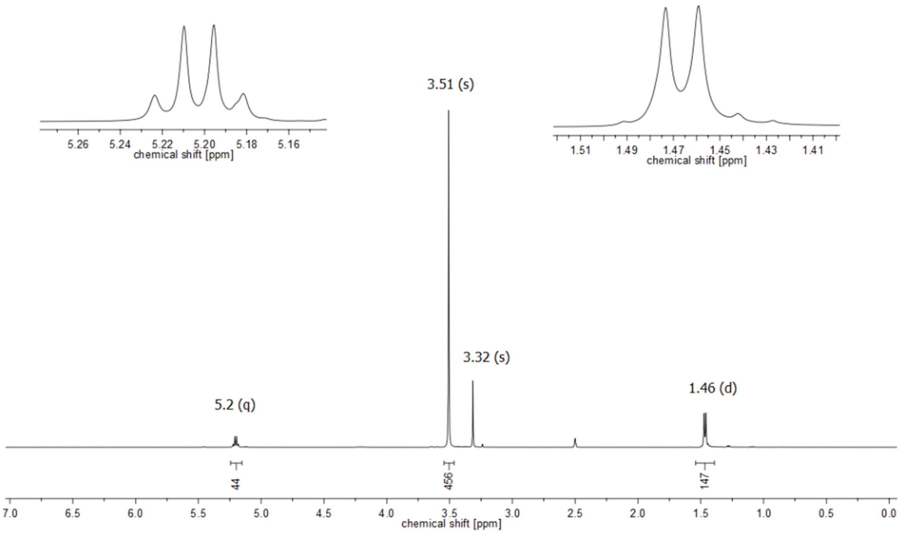

Figure S3: ^{1}H-NMR Spectrum of PEG_{114}-*b*-PLA_{48} solvent (DMSO-d_5).

^{1}H-NMR (500 MHz DMSO-d_6) δ (ppm) = 5.20 (q, ^{3}J = 7.0 Hz, 44H, $CH_3CH(C{=}O)O$ (PLA)), 5.01 (q, ^{3}J =J 7.1 Hz,1H, $CH_3CH(C{=}O)O$ (monomer)), 3.51 (s, 456H, OCH_2CH_2O (PEG)), 3.32 (s, 78H, H_2O), 3.31 (s, 3H, OCH_3) (PEG-OMe)), 1.46 (d, ^{3}J = 7.0 Hz, 147 H, $CH_3CH(C{=}O)O$ (PLA)).

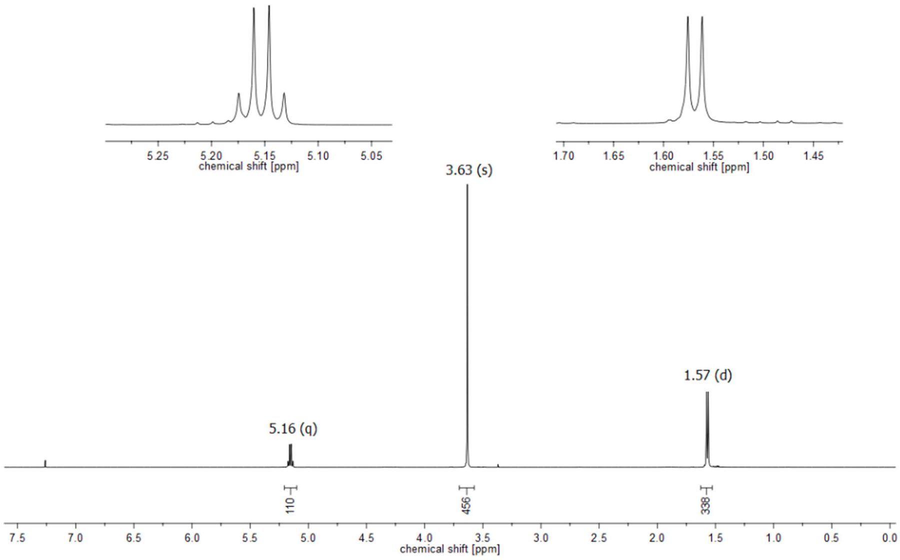

Figure S4: ^{1}H-NMR spectrum of PEG$_{114}$-*b*-PLA$_{112}$ (solvent $CDCl_3$)..

^{1}H-NMR (500 MHz, $CDCl_3$):δ (ppm) = 7.26 (s, $CDCl_3$): 5.16 (q, ^{3}J =J 7.1 Hz, 110H, $CH_3CH(C{=}O)O$ (PLA)), 3.63 (s, 456H, OCH_2CH_2O (PEG)), 3.36 (s, 3H, OCH_3) (PEG-OMe)), 1.57 (d, ^{3}J =7.2 Hz, 338H, $CH_3CH(C{=}O)O$ (PLA)) , 1.48 (d, ^{3}J =7.2 Hz, 6H , $CH_3CH(C{=}O)O$ (monomer)).

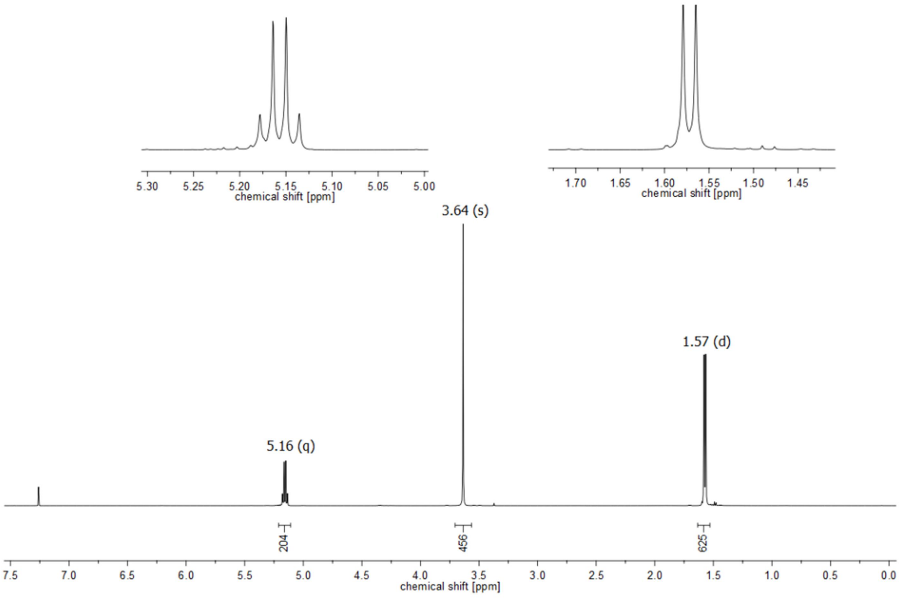

Figure S5: ^{1}H-NMR spectrum of PEG$_{114}$-*b*-PLA$_{207}$ (solvent $CDCl_3$)..

^{1}H-NMR (500 MHz, $CDCl_3$):δ (ppm) = 7.26 (s, $CDCl_3$): 5.16 (q, ^{3}J =J 7.1 Hz, 204H, $CH_3CH(C{=}O)O$ (PLA)), 3.64 (s, 456H, OCH_2CH_2O (PEG)), 3.36 (s, 3H, OCH_3) (PEG-OMe)), 1.57 (d, ^{3}J =7.2 Hz, 625H, $CH_3CH(C{=}O)O$ (PLA)) , 1.48 (d, ^{3}J =7.2 Hz, 10H , $CH_3CH(C{=}O)O$ (monomer)).

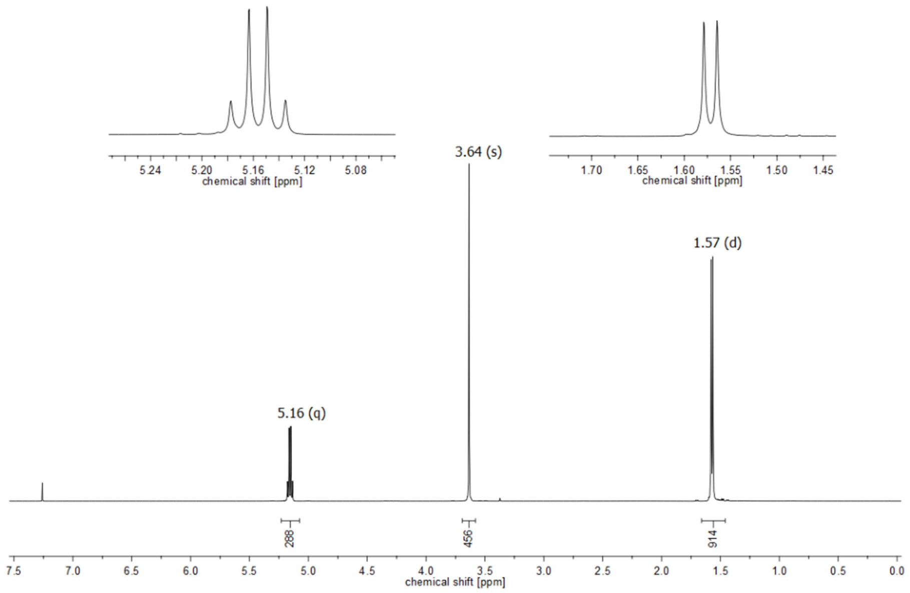

Figure S6: ^{1}H-NMR Spectrum of PEG_{114}-*b*-PLA_{301} (solvent $CDCl_3$).

^{1}H-NMR (500 MHz, $CDCl_3$):δ (ppm) = 7.26 (s, $CDCl_3$): 5.16 (q, ^{3}J =J 7.1 Hz, 288H, $CH_3CH(C{=}O)O$ (PLA)), 3.64 (s, 456H, OCH_2CH_2O (PEG)), 3.36 (s, 3H, OCH_3) (PEG-OMe)), 1.57 (d, ^{3}J =7.2 Hz, 914H, $CH_3CH(C{=}O)O$ (PLA)) , 1.48 (d, ^{3}J =7.2 Hz, 10H , $CH_3CH(C{=}O)O$ (monomer)).

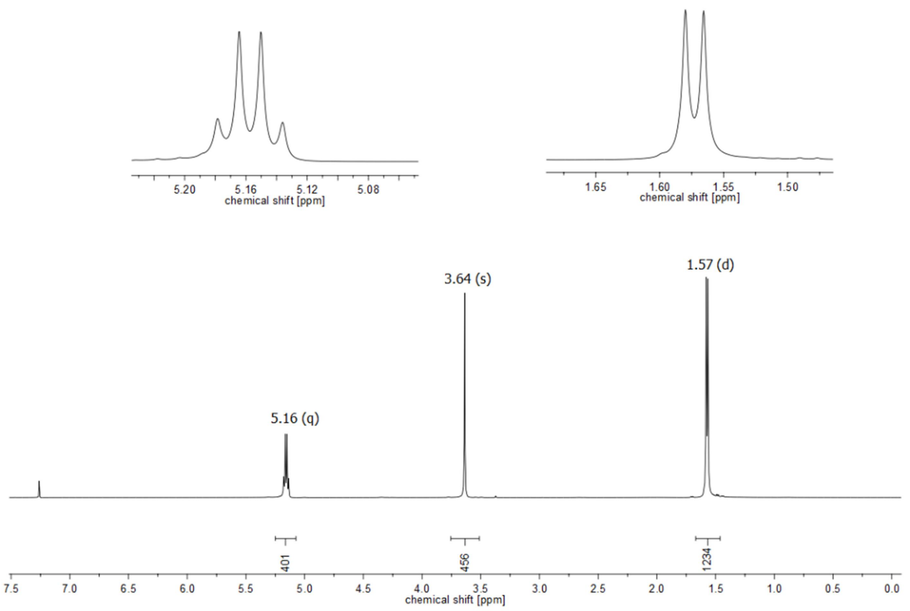

Figure S7: ^{1}H-NMR Spectrum of PEG_{114}-*b*-PLA_{408} (solvent $CDCl_3$).

^{1}H-NMR (500 MHz, $CDCl_3$):δ (ppm) = 7.26 (s, $CDCl_3$): 5.16 (q, ^{3}J =J 7.1 Hz, 401H, $CH_3CH(C{=}O)O$ (PLA)), 3.64 (s, 456H, OCH_2CH_2O (PEG)), 3.36 (s, 3H, OCH_3) (PEG-OMe)), 1.57 (d, ^{3}J =7.2 Hz, 1234H, $CH_3CH(C{=}O)O$ (PLA)) , 1.48 (d, ^{3}J =7.2 Hz, 16H , $CH_3CH(C{=}O)O$ (monomer)).

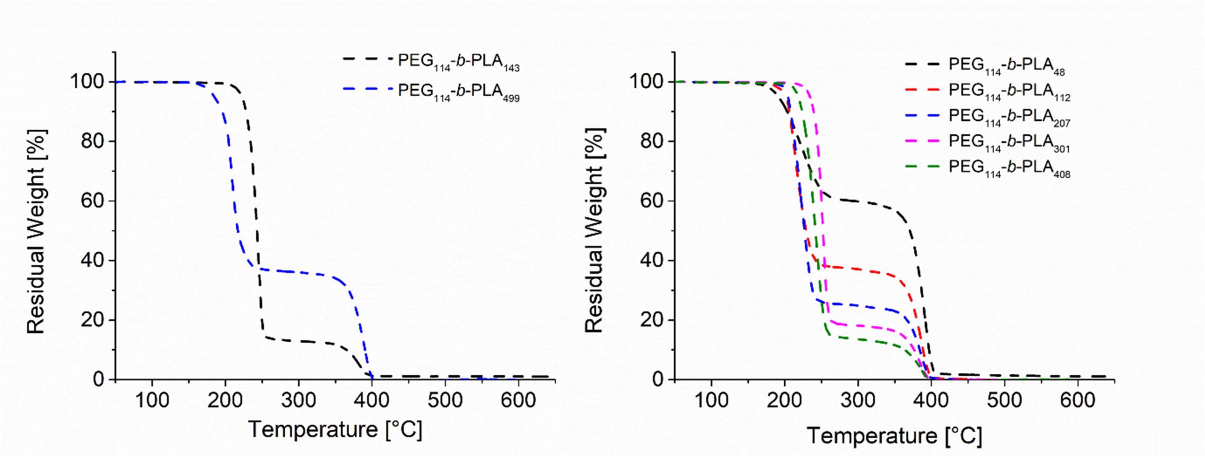

Figure S8: TGA traces of the synthesized PEG_{114}-*b*-PLA_X polymers with a heating rate of 5°C/min from 200 to 450°C under N_2 atmosphere.

Table S1: Values of degree of polymerization of the PLA block of the synthesized PEG_{114}-*b*-PLA_X polymers using ^{1}H-NMR and by weight determined with thermogravimetric analysis (TGA)

**Polymers and nominal degree of polymerization**	**Number average degree of polymerization (^{1}H-NMR spectroscopy)**	**Approximate mass average degree of polymerization (TGA)**
PEG_{114}-*b*-PLA_{150}	143	139
PEG_{114}-*b*-PLA_{500}	499	491
PEG_{114}-*b*-PLA_{50}	48	47
PEG_{114}-*b*-PLA_{100}	112	100
PEG_{114}-*b*-PLA_{200}	207	202
PEG_{114}-*b*-PLA_{300}	301	306
PEG_{114}-*b*-PLA_{400}	408	400

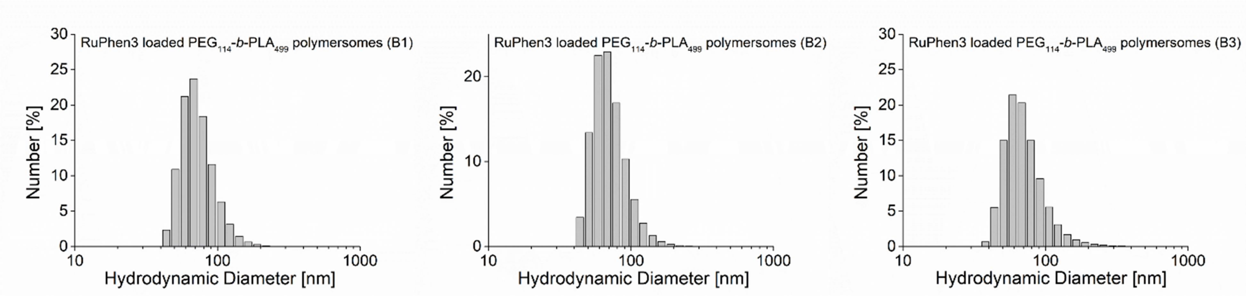

Figure S9: Size Distributions (DLS) of PEG_{114}-*b*-PLA_{499} polymersomes in water (Batches 1-3).

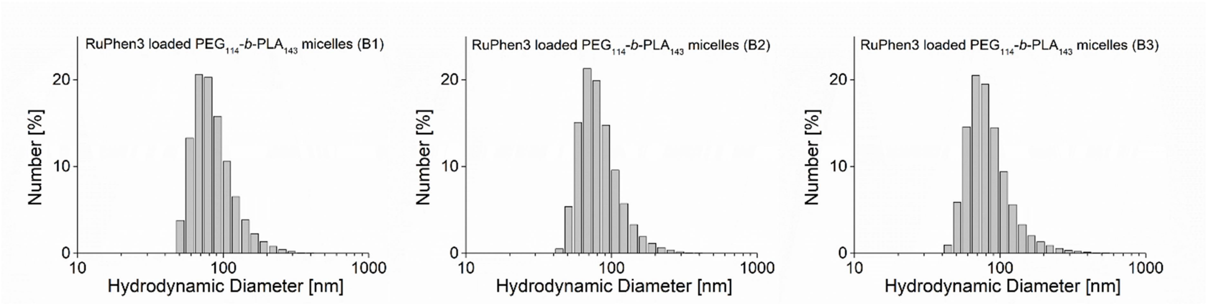

Figure S10: Size Distributions (by DLS) of PEG_{114}-*b*-PLA_{143} micelles in water (Batches 1-3).

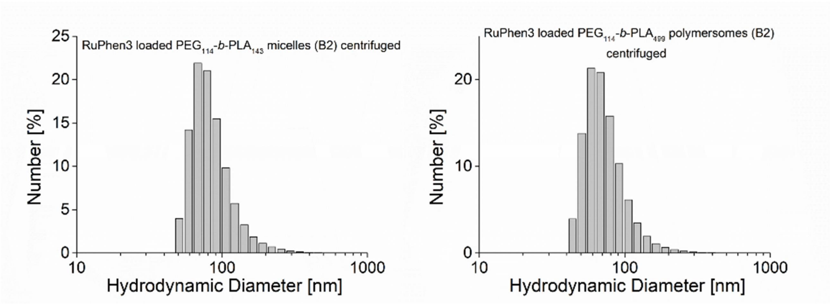

Figure S11: Size Distributions (by DLS) of PEG_{114}-*b*-PLA_{143} micelles and PEG_{114}-*b*-PLA_{499} polymersomes after 10x centrifugation and solvent exchange in water (Batch 2).

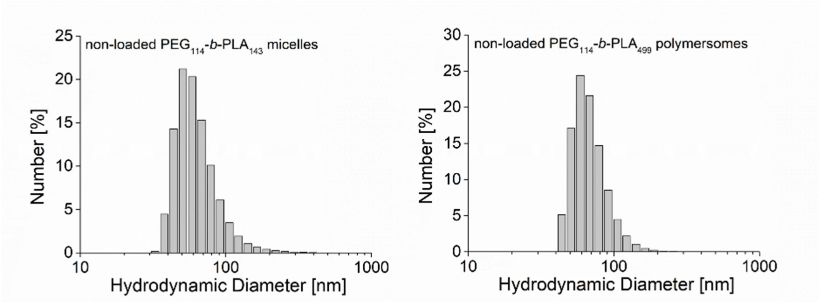

Figure S12: Size Distributions (by DLS) of non-loaded PEG_{114}-*b*-PLA_{143} micelles and PEG_{114}-*b*-PLA_{499} polymersomes in water.

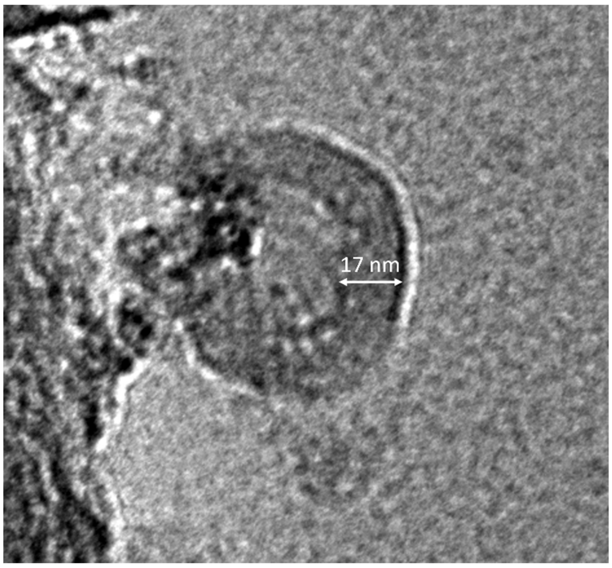

Figure S 13: TEM images of RuPhen3-loaded PEG_{114}-*b*-PLA_{499} polymersomes as shown in Figure 3 with indicated wall thickness (compare arrow).

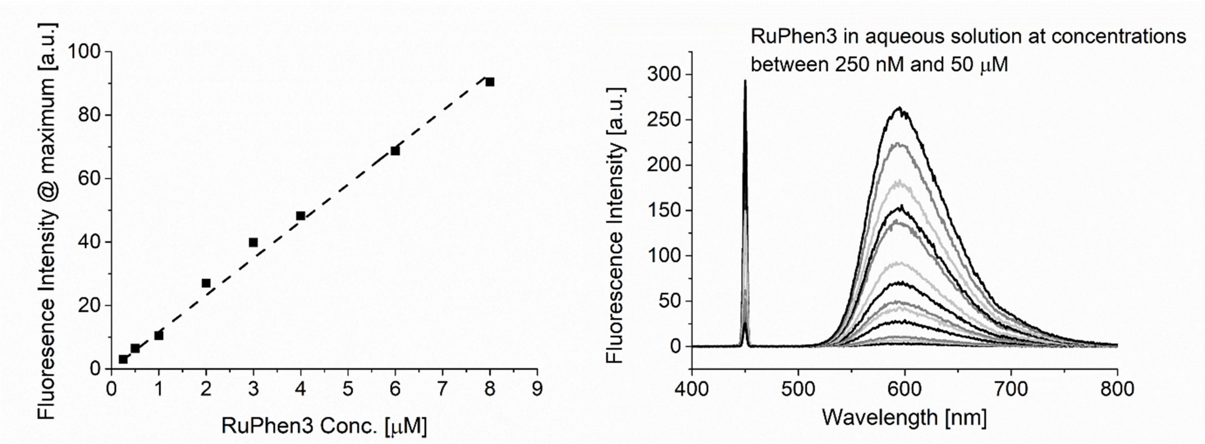

Figure S14: Fluorescence intensity at the maximum emission for different RuPhen3 concentrations in aqueous solution as a function of concentration (left) and fluorescence emission spectra of RuPhen3 in aqueous solution for concentrations between 250 nM and 50 µM (right) excited at 450 nm.

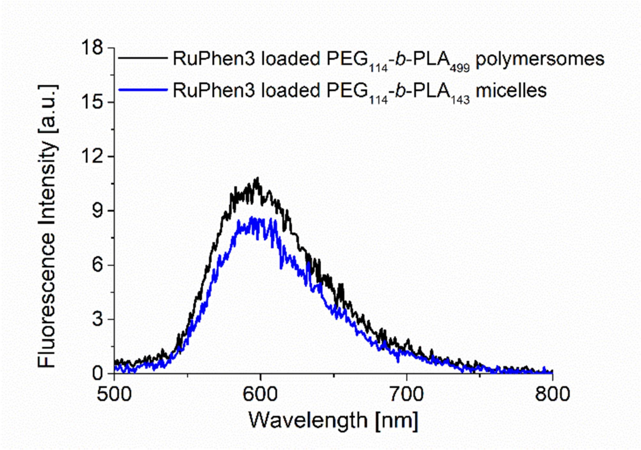

Figure S15: Fluorescence emission spectra of RuPhen3 loaded PEG_{114}-*b*-PLA_{143} micelles and PEG_{114}-*b*-PLA_{499} polymersomes in aqueous solution (Batch3) for upscaled batches excited at 450 nm.

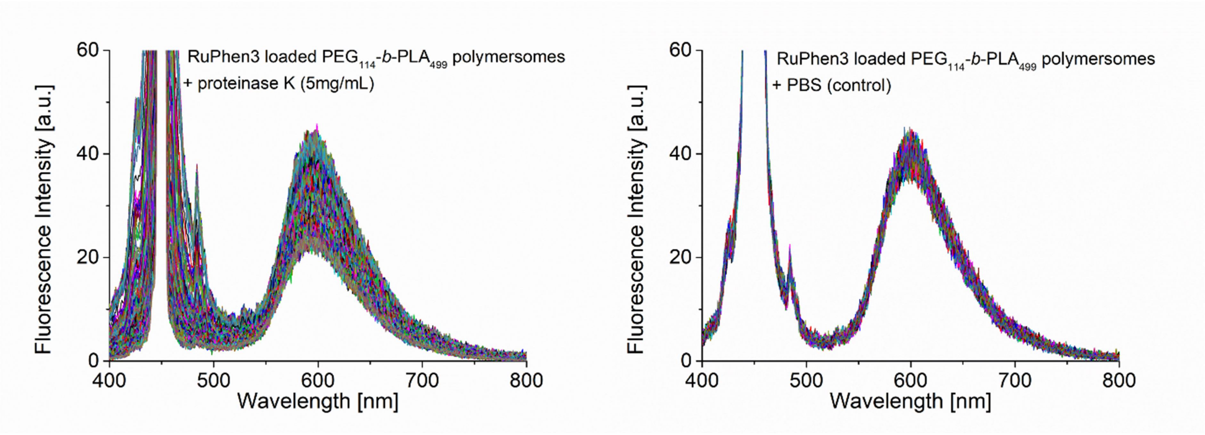

Figure S16: Fluorescence emission spectra of RuPhen3 (excited at 450 nm) encapsulated into PEG_{114}-*b*-PLA_{499} polymersomes over minimum 12h in presence of proteinase K (5mg/mL) in PBS (left) and without addition of enzyme in PBS (right). The spectra show a slow increase followed by strong decrease in fluorescence intensity at around 600 nm (RuPhen3).

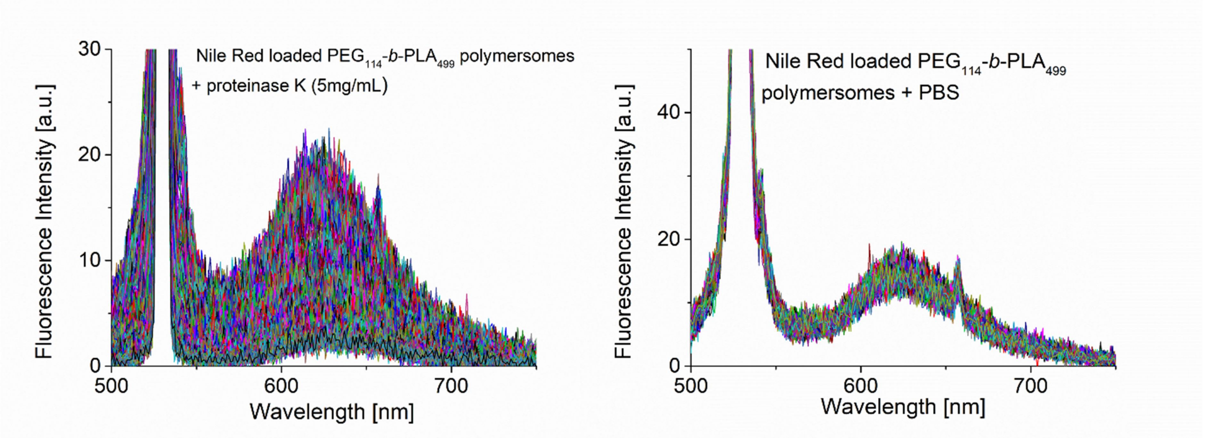

Figure S17: Fluorescence emission spectra of Nile Red (NR)(excited at 530 nm) encapsulated into PEG_{114}-*b*-PLA_{499} polymersomes over minimum 12h in presence of proteinase K (5mg/mL) in PBS (left) and without addition of enzyme in PBS (right). The spectra show a slow increase followed by strong decrease in fluorescence intensity at around 620 nm (NR).

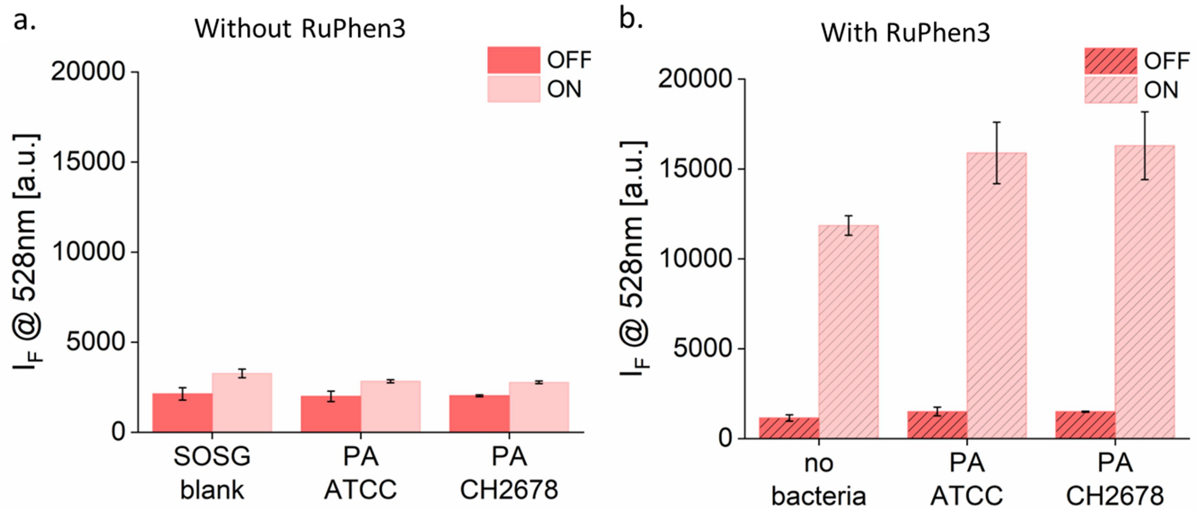

Figure S18: SOSG probe signal for detecting singlet oxygen production by a. SOSG probe and the used bacterial strains PA ATCC and PA CH2678, b. neat RuPhen3 incubated with and without bacteria strains. The samples were irradiated for 30 min (ON) or incubated in dark for 30 min (OFF).

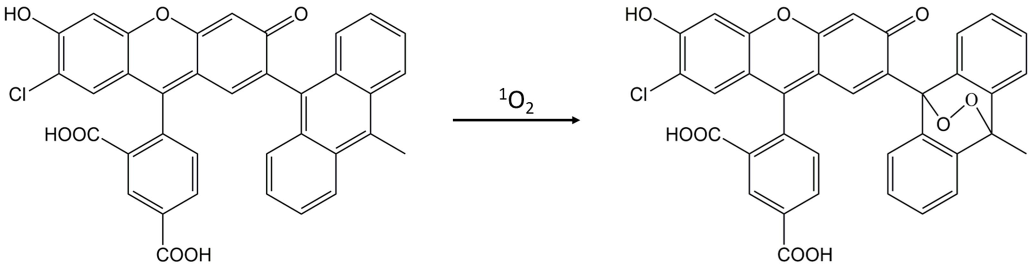

SOSG

Endoperoxide of SOSG

Figure S19*:* The conversion of the SOSG probe into its endoperoxide form in the presence of singlet oxygen.
